# Substrate specificity of human metallocarboxypeptidase D: Comparison of the two active carboxypeptidase domains

**DOI:** 10.1371/journal.pone.0187778

**Published:** 2017-11-13

**Authors:** Javier Garcia-Pardo, Sebastian Tanco, Lucía Díaz, Sayani Dasgupta, Juan Fernandez-Recio, Julia Lorenzo, Francesc X. Aviles, Lloyd D. Fricker

**Affiliations:** 1 Institut de Biotecnologia i Biomedicina and Departament de Bioquimica i Biologia Molecular, Universitat Autònoma de Barcelona, Bellaterra, Barcelona, Spain; 2 VIB-UGent Center for Medical Biotechnology, Ghent, Belgium; 3 Department of Biochemistry, Ghent University, Ghent, Belgium; 4 Barcelona Supercomputing Center (BSC), Joint BSC-CRG-IRB Research Program in Computational Biology, Life Sciences Department, Barcelona, Spain; 5 Department of Molecular Pharmacology, Albert Einstein College of Medicine, Bronx, New York, United States of America; Russian Academy of Medical Sciences, RUSSIAN FEDERATION

## Abstract

Metallocarboxypeptidase D (CPD) is a membrane-bound component of the trans-Golgi network that cycles to the cell surface through exocytic and endocytic pathways. Unlike other members of the metallocarboxypeptidase family, CPD is a multicatalytic enzyme with three carboxypeptidase-like domains, although only the first two domains are predicted to be enzymatically active. To investigate the enzymatic properties of each domain in human CPD, a critical active site Glu in domain I and/or II was mutated to Gln and the protein expressed, purified, and assayed with a wide variety of peptide substrates. CPD with all three domains intact displays >50% activity from pH 5.0 to 7.5 with a maximum at pH 6.5, as does CPD with mutation of domain I. In contrast, the domain II mutant displayed >50% activity from pH 6.5–7.5. CPD with mutations in both domains I and II was completely inactive towards all substrates and at all pH values. A quantitative peptidomics approach was used to compare the activities of CPD domains I and II towards a large number of peptides. CPD cleaved C-terminal Lys or Arg from a subset of the peptides. Most of the identified substrates of domain I contained C-terminal Arg, whereas comparable numbers of Lys- and Arg-containing peptides were substrates of domain II. We also report that some peptides with C-terminal basic residues were not cleaved by either domain I or II, showing the importance of the P1 position for CPD activity. Finally, the preference of domain I for C-terminal Arg was validated through molecular docking experiments. Together with the differences in pH optima, the different substrate specificities of CPD domains I and II allow the enzyme to perform distinct functions in the various locations within the cell.

## Introduction

Metallocarboxypeptidases (MCPs) are zinc-dependent enzymes that cleave single amino acids from the C termini of peptides and proteins [1,2]. The first MCP to be identified was carboxypeptidase A1 (CPA1), a pancreatic enzyme that removes C-terminal hydrophobic residues. In the ensuing decades since the discovery of CPA1, dozens of additional MCPs have been identified based on sequence and/or structural similarity; these are classified as M14 family enzymes by MEROPS [3]. All mammalian MCPs show marked preference for the residue in the P1’ site, and are selective for either hydrophobic residues, basic residues, or acidic residues [1,4]. Several M14 gene family members are not active towards conventional carboxypeptidase substrates, although it remains possible that they hydrolyze atypical substrates [5–7]. Most of the MCPs show differential patterns of expression. The combination of substrate specificity and distribution are responsible for the diverse functions of these proteins which ranges from digestion of food (for carboxypeptidases A1, A2, and B1) to the production of neuropeptides and peptide hormones (carboxypeptidase E) and the selective processing of tubulin (cytosolic carboxypeptidases) [1,8].

**Fig 1:**
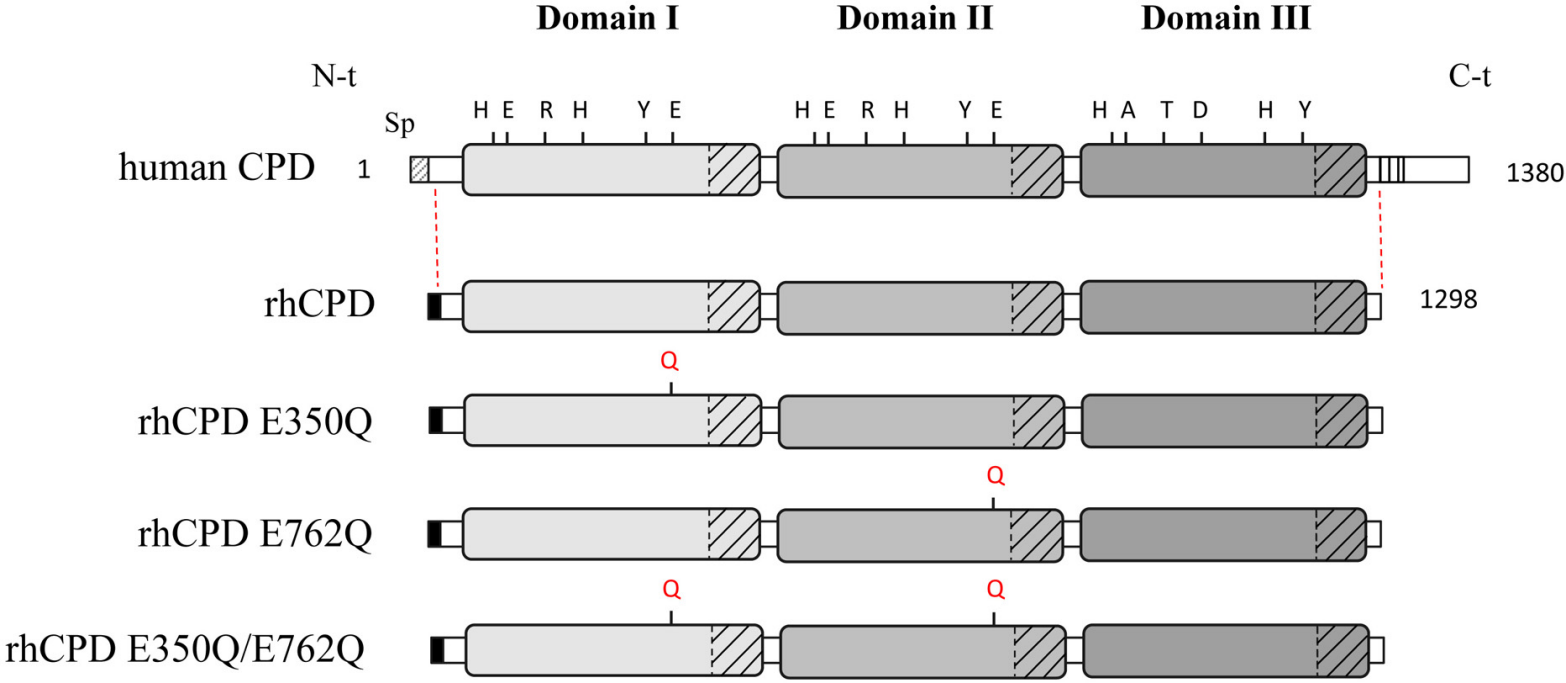
Linear representation of full-length human CPD and recombinant forms showing the location of single point mutations. The positions indicated in human CPD correspond to key residues essential for the catalytic mechanism: His69, Glu72, Arg145, His198, Tyr248, and Glu270 (according to the bCPA1 numbering). Sp, indicates the location of the endogenous signal peptide on the N-terminus. Recombinant proteins correspond to C-terminal truncated forms of human CPD, which lack the C-terminal transmembrane anchor and the cytoplasmic tail. The mutations (*i*.*e*., Glu to Gln) performed to generate single point mutants for the CPD domain I (E350Q), domain II (E762Q) and a double mutant (E350Q/E762Q) are indicated. The black shaded box on the N-term of the recombinant proteins corresponds to the IgM secretion signal sequence and N-terminal Strep-Tag II fusion protein.

Carboxypeptidase D (CPD) belongs to the M14B subfamily of MCPs and was originally discovered in duck hepatocytes as a 180-kDa membrane-bound glycoprotein (named gp180) that has the ability to bind duck hepatitis B virus particles [9,10]. Bovine CPD was independently discovered in a search for an enzyme with carboxypeptidase E-like properties [11]. In all species examined, CPD was found in all tissues examined, displaying a broad tissue distribution [12–14]. Except for CPD, all other members of the M14B subfamily contain a single domain of ~300 amino acids with homology to CPA1 (and other enzymes in the M14A subfamily) followed by ~80 amino acids with structural homology to transthyretin. In contrast, CPD from mammals, birds, and *Drosophila* contain three of these carboxypeptidase/transthyretin-like domains (**Fig 1**) [10,11,15,16].

The critical catalytic residues, metal-binding residues, and substrate-binding residues found in all active members of the M14 gene family are conserved in CPD domains I and II but not in domain III. CPD also contains a transmembrane segment of about 20 residues and a C-terminal cytosolic tail of 60 residues (**Fig 1**). CPD is primarily localized in the trans-Golgi network (TGN), and cycles between the TGN and the cell surface [17]. The 60-residue cytosolic tail has previously been shown to be both necessary and sufficient for the trafficking of CPD, and sequences within this region have been found to bind cytosolic proteins (such as protein phosphatase 2A) and mediate TGN retention and intracellular trafficking [18–20].

The three carboxypeptidase (CP) domains of CPD are localized to the lumen of the TGN, exocytic vesicles, and endocytic vesicles, and are exposed to the extracellular environment when the enzyme is transiently present on the cell surface [21]. Based on the broad tissue distribution of CPD, its subcellular localization, and ability to cleave basic residues from several peptides that have been tested, CPD is thought to play a role in the further processing of proteins and peptides that are initially cleaved by furin and furin-like enzymes within the secretory pathway, on the cell surface, and within the endocytic pathway. Previous studies testing a very small number of synthetic substrates found that duck and *Drosophila* CPD domains I and II are enzymatically active, but the third domain of CPD is catalytically inactive toward traditional MCP substrates [16,22]. Duck and *Drosophila* CPD domain I are optimally active at neutral pH and cleave a peptide with C-terminal arginine more efficiently than a similar peptide with C-terminal lysine [16,22]. By contrast, CPD domain II is optimally active at slightly acidic pH and cleaves a peptide with C-terminal lysine as efficiently as a peptide with C-terminal arginine [9,16,22]. However, these previous studies tested only one peptide with C-terminal Lys, and it was not clear if these results could be generalized to all Lys-containing peptides. Sidyelyeva *et al*. investigated the function of the various CP domains through the analysis of spontaneous mutations in the various domains and through the creation of flies expressing specific forms of CPD in the svrPG33 mutant [23,24]. All mutants containing an active CP domain rescued the lethality with varying degrees, in all cases requiring the presence of inactive CPD domain III for full viability. Transgenic flies expressing active CPD domain I or domain II showed similar behaviors to each other and to the viable svr mutants, suggesting redundant functions in terms of processing peptides involved in viability and in behaviors like cold and ethanol sensitivity, as well as long-term memory [24].

CPD has been implicated in several human diseases. In one study, CPD was found to be downregulated in CD14 positive cells isolated from patients with lupus erythematosus [25]. In a study using high-density oligonucleotide microarrays with colon biopsies, CPD was identified as one of seven discriminatory genes that could functionally classify patient samples as adenoma, colorectal carcinomas or inflammatory bowel diseases [26]. CPD is frequently overexpressed in human cancers such as hepatocellular carcinoma, with 164 of 400 samples showing high expression of CPD; downregulation of CPD inhibited carcinoma cell proliferation through induction of G1 cell-cycle arrest and apoptosis [27]. In a study of human prostate specimens, CPD immunostaining was higher in cancer cells than in benign cells [28].

Despite the numerous studies finding CPD to be associated with human disease, the enzymatic properties of human CPD have not been studied in detail. The objective of the present study was to investigate the properties of human CPD and functionally characterize its individual catalytic domains. For this, we generated catalytically inactive single point mutants for the individual active domains I and II (E350Q, E762Q, respectively) as well as a double mutant (**Fig 1**). We characterized the enzymatic properties of the full active human CPD and the individual catalytically active domains, using a wide range of substrates and quantitative peptidomics approaches. We found that human CPD domain III is inactive towards all peptides examined, while domains I and II have complementary activities that provide for a wider ability to cleave peptides/proteins with basic C-terminal residues within the secretory pathway, on the cell surface, and within the endocytic pathway. However, not all peptides with C-terminal basic residues were efficiently cleaved by domains I and/or II, indicating the importance of other residues within the peptide. Finally, we modeled the structure of all three domains of hCPD and performed molecular docking experiments with a set of peptide substrates. Our thorough characterization of the substrate specificities of the individual domains of human CPD suggests the functional specialization of each CPD domain to distinct subcellular environments.

## Materials and methods

### Cell culture

HEK293T cells (ATCC CRL-3216) were cultured in Dulbecco’s Modified Eagle’s Medium (DMEM) supplemented with GlutaMAX and 10% (v/v) fetal bovine serum (Invitrogen, Inc.) at 37°C, 10% CO_2_ and 95% humidity. HEK293F cells (Free Style^TM^ 293-F Cells, Thermo-Fischer Scientific) were grown in FreeStyle 293 Expression Medium (Invitrogen, Inc.) in flasks on a rotary shaker (120 rpm) at 37°C, 8% CO_2_ and 70% humidity.

### Recombinant protein production and purification

Human carboxypeptidase D (residues 32–1298) was cloned into the pTriEx^TM^-7 expression vector (Merck Millipore) encoding for mouse IgM secretion signal sequence and an N-terminal Strep-Tag II fusion protein. CPD E270Q mutants (according to bovine CPA1 numbering) named here as E350Q, E762Q (for single mutants) and E350Q/E762Q (for the double mutant) were generated by PCR-driven overlap extension [29]. For protein production in mammalian cells, DNA transfections of CPD and the CPD E270Q mutants were carried out using 25-kDa polyethylenimine (Polysciences) in a ratio of 1:3 (μg DNA/μg polyethylenimine), as described previously [30]. Briefly, HEK293F cells were diluted to a cell density of about 0.5x10^6^ cells/ml, grown in FreeStyle 293 Expression Medium for 24 h and then transfected with 1 μg DNA per ml of culture for 7 days. For protein purification, 500 ml of the culture supernatant were equilibrated with 30% ammonium sulphate, centrifuged, filtered through 0.2 μm filters and bound to a 15 ml hydrophobic chromatography column (Toyopearl Butyl-650M, Tosoh Bioscience). Protein elution was carried out with a decreasing gradient of ammonium sulphate (30 to 0%) in a 100 mM Tris-HCl, pH 7.5 buffer. The eluted fractions containing CPD (as analyzed by SDS-PAGE) were pooled and loaded onto a 5 ml Strep-Tactin affinity column (IBA GmbH) equilibrated with 5 column volumes (cv) binding buffer (100 mM Tris-HCl, pH 8.0, 150 mM NaCl), washed with 2 cv of binding buffer and eluted with 8 cv of the elution buffer (*i*.*e*.,binding buffer containing 2.5 mM *d*-desthiobiotin; IBA GmbH). Eluted fractions were analyzed by SDS-PAGE, and the purest fractions were pooled and loaded onto a HiLoad Superdex 75 26/60 column (GE Healthcare) previously equilibrated with a 50 mM Tris-HCl, pH 7.5, 150 mM NaCl buffer. The purified proteins were flash frozen at a concentration of approximately 0.5 mg/ml and stored at -80°C.

### HEK293T bortezomib treatment and peptide extraction

HEK293T cells were seeded in 24 150-mm cell culture plates and after growing up to 70% confluence, cells were treated with fresh media containing 0.5 μM bortezomib for 1 h. Following incubation, cells were washed three times with cold Dulbecco’s phosphate-buffered saline (DPBS, Invitrogen) supplemented with 0.5 μM bortezomib, immediately scraped and centrifuged at 8000xg for 5 min. For peptide extraction, the cell pellet was resuspended in 1 ml of 80°C water and the mixture was incubated for 20 min in an 80°C water bath. Samples were cooled, transferred to 2 ml low retention microfuge tubes and centrifuged at 13,000xg for 20 min. Soluble fractions containing HEK293T cell peptides were stored at -80°C overnight. Samples were centrifuged again and the supernatant of each tube was collected and concentrated in a vacuum centrifuge to a volume of 1.5 ml. Finally, samples were cooled and acidified with 0.1 M HCl to get a final concentration of 10 mM HCl. After 15 minutes of incubation, samples were centrifuged at 13000xg for 40 min at 4°C and the supernatants stored at -80°C until labelling.

### Preparation of tryptic peptide libraries

Tryptic peptide libraries were generated by digesting five different proteins with trypsin. Trypsin is an endoprotease that cleaves C-terminal to arginine or lysine residues, producing peptides containing basic residues on their C-termini [31]. The proteins bovine serum albumin (BSA), bovine thryroglobulin, bovine α-lactalbumin, human α-hemoglobin and human β-hemoglobin (all from Sigma-Aldrich) were digested separately and then pooled in order to have a tryptic peptide library with a final peptide concentration of about 500 μM. To prepare the tryptic peptide libraries, 5 nmoles of each protein were digested for 16 h at 37°C in a 20 mM borate, pH 7.6 buffer using sequencing-grade trypsin (Promega) at an enzyme/substrate ratio of 1/100 (w/w). The efficiency of protein digestion was checked by SDS-PAGE. To stop the proteolytic digestion, 100 mM HCl was added to a final concentration of 1 mM. All the reactions were combined and centrifuged at 13,000xg for 45 min at 4°C. The supernatant containing tryptic peptides was filtered through a 10-kDa centrifugal filter device (Amicon, Merck Millipore), aliquoted and stored at -80°C until use.

### Kinetic measurements using fluorescent substrates

Carboxypeptidase activity was assayed with the fluorescent substrates dansyl-Phe-Ala-Arg, dansyl-Phe-Gly-Arg or dansyl-Phe-Pro-Arg synthesized as described [32]. The assay was modified slightly from the previously described procedure. In brief, 0.2 mM of substrate was incubated with enzyme at the indicated concentrations (see **S1 Fig**) for 60 min at 37°C in 100 μl of 100 mM Tris-acetate (pH 6.4) containing 100 mM NaCl. Reactions were stopped by adding 50 μl of 500 mM HCl and 1 ml of chloroform. Tubes were mixed and centrifuged for 2 min at 300xg. After centrifugation, 0.5 ml of the chloroform phase was transferred to new tubes and dried overnight at 25°C in a fume hood. Dried samples were resuspended with 200 μl of PBS containing 0.1% of Triton X-100. The amount of product generated was determined by measuring the fluorescence emission of samples at 495 nm upon excitation at 350 nm using a 96-well plate spectrofluorometer. For kinetic analysis, purified enzymes (at different concentrations, i.e. 2.5 nM for the full-active enzyme; 5.0 nM for CPD E762Q; 20.0 nM for CPD E350Q; and 100.0 nM for CPD E350Q/ E762Q were assayed at different substrate concentrations (6.25, 12.5, 25, 50, 100, 200, 300 and 600 μM). In all cases, a maximum of 20% of the substrate was hydrolyzed. Kinetic parameters were determined by fitting the obtained data for each enzyme to the Michaelis-Menten equation (y = (*V*_max_ x X) /(*K*_m_ + X)) using GraphPad Prism software [33], where X is the substrate concentration, *V*_max_ is the maximum enzyme velocity and *K*_m_ is the Michaelis-Menten constant. The pH optimum of purified enzymes was determined with 0.2 mM dansyl-Phe-Ala-Arg in 100 mM Tris-acetate, 150 mM NaCl buffer at the indicated pH.

### Peptidomics

Quantitative peptidomics experiments were performed as described previously [34,35], with slight modifications. We used the peptide library generated using trypsin or by peptide extraction from HEK293T cells, as described above. The peptide mix was incubated for 16 h at 37°C in 100 mM borate, pH 6.5, 100 mM NaCl buffer with different amounts (0, 1, 10 and 100 nM) of purified rhCPD. In a second round of experiments, peptides from the peptide library generated with trypsin were incubated with rhCPD and with the CPD single point mutants (E350Q, E762Q and E350Q/E762Q) at a concentration of 100 nM for 16 h at 37°C. After incubation, reactions were quenched and peptides were labeled using 4-trimethylammoniumbutyrate isotopic tags activated with N-hydroxysuccinimide (TMAB-NHS) containing either all hydrogen (D0), 3 deuteriums (D3), 6 deuteriums (D6), 9 deuteriums (D9), or 9 deuteriums and three atoms of ^13^C (D12). The labels were dissolved in DMSO to a concentration of 0.4 mg/μl and 5 mg of label was used per sample. At the start of the experiment, the pH of the sample was adjusted to 9.5 with 1M NaOH. Labeling was performed over 8 rounds; 1.6 μl of the label was added to the extract every 20 min. The pH was measured between each round and if necessary, brought back to 9.5, only for the first five rounds. After labeling, 30 μl of 2.5 M glycine was added to quench any unreacted label. Labeled samples for a single experiment were pooled, filtered through a10-kDa centrifugal filter device and 30 μl of 2M hydroxylamine was added to hydrolyze any labeled tyrosines. This was done to ensure that only N-terminal amines and lysine side-chain amines of peptides are TMAB-labeled and not tyrosines. Samples were desalted through C-18 spin columns (Thermo-Scientific) by following the manufacturer’s instructions. Peptides were eluted using 160 μl of 0.5% TFA and 70% acetonitrile, freeze-dried in a vacuum centrifuge, and subjected to liquid chromatography and mass spectrometry as described [34,35]. Identifications were performed using Mascot software (Matrix Science). The MS spectra were manually examined for peak sets reflecting peptides with the various isotopic forms of the TMAB tags. Identifications were rejected unless 80% or more of the major fragments from the MS/MS matched predicted b- or y-series fragments (with a minimum of five matches). Additional criteria followed to consider the matches includes the coincidence with the parent mass within 50 ppm of the theoretical mass, an expected charge equal to basic residues plus the N-terminus and a correct number of isotopic tags incorporated considering the number of free amines present in the peptide. Peptidomics analyses were performed at least in triplicate.

### Structural alignment, homology modeling and surface representations

The amino acid sequences of human CPD, as well as human CPM and bovine CPA were obtained from the UniProt database (accession codes O75976, P14384 and P00730, respectively). A structure-based sequence alignment between CPD domains I, II and III was generated using PROMALS3D multiple sequence and structure alignment server [36].

Structural models of the different human CPD domains were constructed using the Prime Homology Modeling Workflow implemented in Schrödinger and further subjected to an energy-based refinement to improve their quality [37,38]. The structure of duck CPD (PDB: 1H8L) was taken as a template, based on its higher sequence identity with the targets (*i*.*e*., 38% SI with hCPD domain I; 79% SI with domain II and 31% SI with domain III) (See multiple alignment from **S3 Fig**). Final models contained the catalytic zinc ion, which was transferred from the 1H8L structure. Electrostatic potential calculations for the three domains, including the zinc ion, were carried out using APBS (Adaptive Poisson-Boltzmann Solver) and the corresponding molecular surface represented with PyMOL [39].

The substrate binding sites of the domains were further characterized with SiteMap [40] by using a grid of points able to identify hydrophobic and hydrophilic regions, hydrogen bond donor, hydrogen bond acceptor and metal-binding regions.

### Peptide docking studies

The peptide docking was performed using the standard-precision docking protocol for peptides from Glide [41]. Ten million poses were initially kept and 1,000 poses per ligand were used for energy minimization. The best 101 docking poses were subjected to full force-field minimization, and these finally resulting poses were selected for further analysis. For the docking, we used the structure of zinc-containing form of the enzyme. However, for practical calculations, the zinc ion was not included during the flexible peptide docking search, given that the empirical force-field used cannot fully describe all the complexities derived from the charge delocalization of the zinc ion, due to its coordination with a water molecule as well as with His and Glu side-chains in MCPs. Nevertheless, the zinc ion was included for visual inspection of the results and in the calculation of the electrostatic and donor/acceptor maps. PyMOL [39] was used for generation of figures.

## Results

### Recombinant protein production and purification

Several expression systems were explored to produce active recombinant CPD. *Pichia pastoris* was previously used to produce CPD domain I and CPD domain II, as well as other members of the M14 MCP family [34,42–44]. However, human CPD containing all three domains did not express well in this system, possibly due to structural complexity and/or the numerous post-translational modifications like N-linked glycosylation. Previous studies produced substantial levels of full-length CPD using the baculovirus expression system [9,22]. However, there are fundamental differences in the glycoprotein processing pathways of insects and higher eukaryotes, which lead to structural and functional differences between native and such recombinantly produced proteins. Recently, mammalian-based expression systems have emerged as promising systems to obtain high levels of complex mammalian proteins by transient or stable transfection [45,46]. For this reason, we cloned residues 32–1298 of human carboxypeptidase D (named here as rhCPD) into the pTriExTM-7 expression vector that encodes an IgM secretion signal sequence and an N-terminal Strep-Tag II fusion protein (**Fig 1**). Residues 32–1298 of CPD encode the three carboxypeptidase-like domains and lack the C-terminal transmembrane domain and cytosolic tail. The CPD-pTrieX-7 construct was transfected into mammalian HEK293F cells for transient expression, using polyethylenimine as transfection reagent. The secreted recombinant protein showed a major band of 170 kDa by western blot analysis using an anti-Strep-tag II antibody, consistent with the predicted molecular weight for rhCPD (data not shown). Maximum levels of soluble rhCPD in the medium were detected 7 days post-transfection.

**Fig 2:**
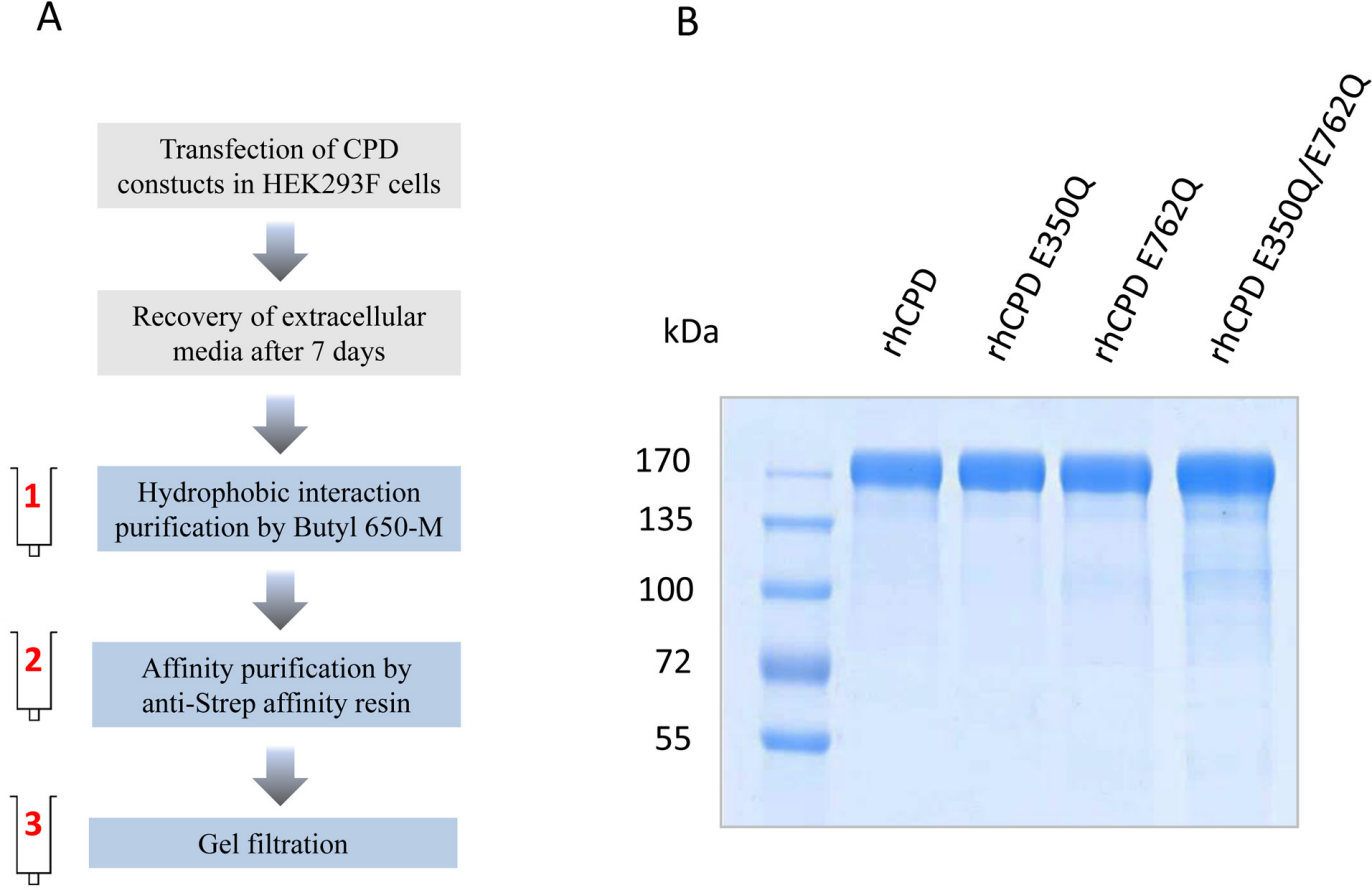
Expression and purification of CPD. (A) Schematic diagram of the strategy for expression and purification of rhCPD and mutants. Protein expression was performed by transient transfection of suspension-growing HEK293F cells. Extracellular medium was collected after 7 days incubation, followed by purification of the recombinant proteins in three steps; (1) hydrophobic interaction chromatography using a Butyl 650-M, (2) affinity chromatography using anti-Strep-tag resin, and (3) gel filtration chromatography. (B) Coomassie-stained SDS–PAGE showing the purity of recombinant CPD proteins.

A variety of conditions were tested to purify rhCPD from the medium, and optimal results were obtained following a three-step chromatography protocol. The first chromatographic step was a hydrophobic interaction chromatography. The eluates containing the rhCPD protein from the first purification step were pooled and purified over an anti-Strep-tag affinity resin and, further fractionated on a size exclusion column (**Fig 2A**) to obtain a high-purity protein product (**Fig 2B**). To characterize the substrate specificity of the individual catalytic domains we generated constructs with single point mutations in domains I or II as well as a double mutant, named here as E350Q, E762Q and E350Q/E762Q, respectively (**Fig 1**). All mutants were expressed and purified following the protocol optimized for rhCPD (**Fig 2B**). In gel filtration chromatography, all mutants eluted in a single peak with comparable apparent masses (data not shown).

### Effect of pH on CPD activity

**Fig 3:**
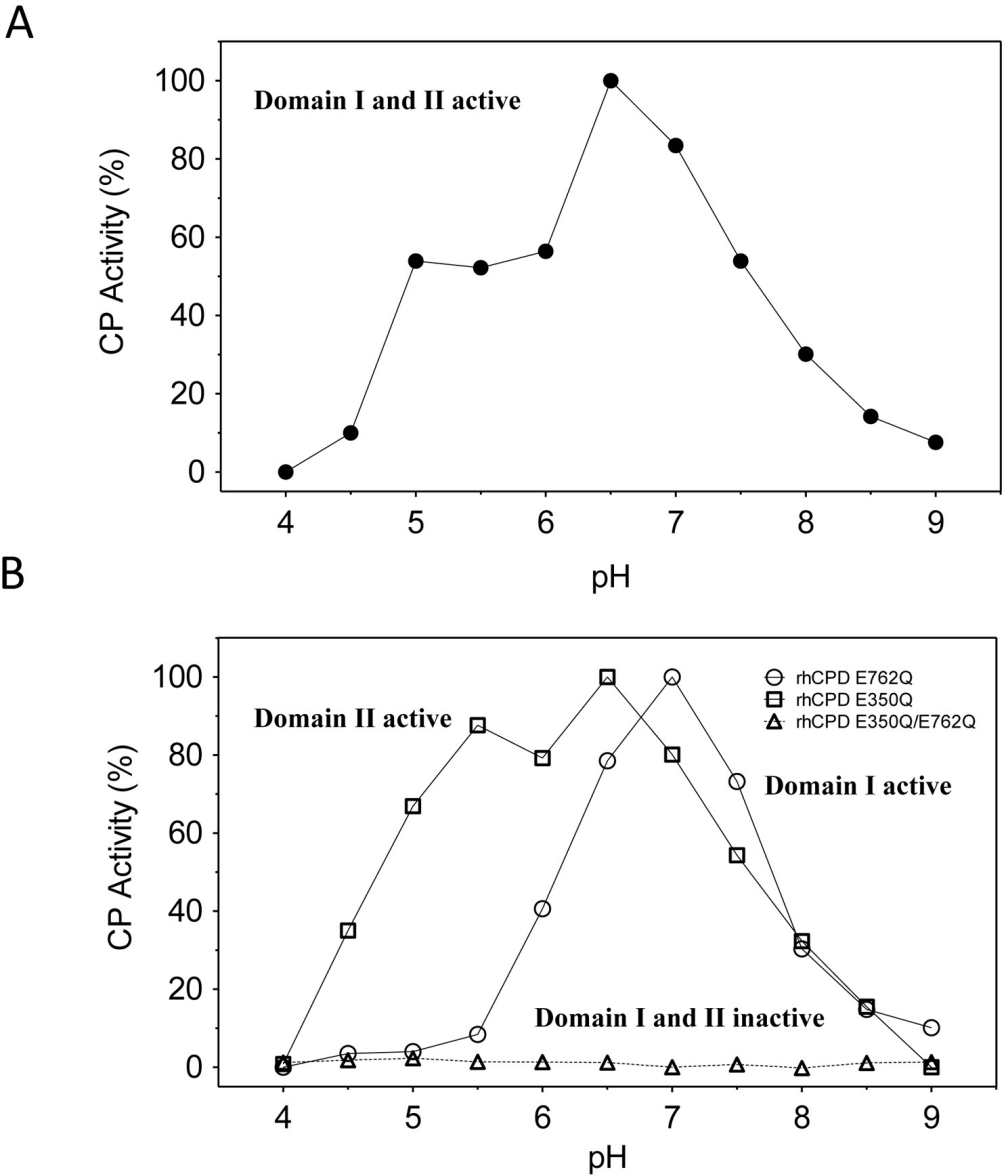
Effect of pH on the activity of different recombinant CPD forms. (A) Effect of pH on rhCPD and (B) single point mutants using 200 μM dansyl-Phe-Ala-Arg in a Tris-acetate buffer at the indicated pH for 60 min at 37°C. The activity represented is the average of three independent measures with less than a 10% of variation, expressed as a percentage of the maximal activity, observed at optimal pH.

The influence of pH on rhCPD and rhCPD single point mutants were examined using the fluorescent substrate dansyl-Phe-Ala-Arg. The pH optimum of the human protein with both domains I and II active is 6.5, with >50% maximal activity from 5.0–7.5 (**Fig 3A**). The construct E350Q shows optimal activity at pH 6.5, with activity >50% of the maximum over the range 5.0–7.5, while the construct E762Q has optimal activity at pH 7.0 and with >50% maximal activity over the range 6.5–7.5 (**Fig 3B**). The double mutant E350Q/E762Q had no detectable enzyme activity at any of the pH values examined (**Fig 3B**).

### Activity of CPD and CPD single point mutants with fluorescent substrates

The enzymatic activities of rhCPD were tested with three fluorescent synthetic substrates. To compare substrates, different amounts of the purified enzyme were incubated with 200 μM of each dansylated peptide and the relative amount of product determined. Among the substrates evaluated, dansyl-Phe-Ala-Arg was the peptide most rapidly cleaved by rhCPD (**S1 Fig**); at enzyme concentrations where 70% of dansyl-Phe-Ala-Arg was cleaved, less than 5% of dansyl-Phe-Gly-Arg was hydrolyzed by the enzyme. No activity was detected towards the substrate with a Pro residue in penultimate position (*i*.*e*., dansyl-Phe-Pro-Arg). We determined the kinetic parameters for dansyl-Phe-Ala-Arg for the full active rhCPD and the single point mutants E350Q and E762Q (**Table 1)**. These studies were conducted at pH 6.5 because all three proteins showed ≥80% maximal activity at this pH value. The *k*_cat_ value of the E350Q mutant with only domain II active (7.0 ± 0.9 s^-1^) is generally comparable to the *k*_cat_ of the E762Q mutant with domain I active (8.5 ± 0.5 s^-1^) and both are smaller than the *k*_cat_ for rhCPD with both active domains (12.5 ± 0.5 s^-1^). The lowest *K*_m_ value was obtained for the full active rhCPD, which showed a *K*_m_ of 153 ± 16 μM, while single point mutants E762Q and E350Q showed higher *K*_m_ values of 319 ± 37 μM and 844 ± 139 μM, respectively. The *k*_cat_/*K*_m_ value for rhCPD was approximately 3 times and 10 times higher in comparison with values obtained for the E762Q and E350Q single point mutants, respectively (**Table 1**).

**Table 1:** Kinetic constants for hydrolysis of dansyl-Phe-Ala-Arg by rhCPD and rhCPD single point mutants.

Substrate	rhCPD	Active domain I rhCPD (E762Q)	Active domain II rhCPD (E350Q)	Double mutant rhCPD (E762Q /E350Q)
Dansyl-Phe-Ala-Arg				
*k*_*m*_ *(μM)*	153 ± 16	319 ± 37	844 ± 139	ND
*k*_*cat*_ *(s*^*-1*^*)*	12.5 ± 0.5	8.5 ± 0.5	7.0 ± 0.9	ND
*k*_*cat*_*/ K*_*m*_ *(μM*^*-1*^ *s*^*-1*^*)*	0.082 ± 0.012	0.027 ± 0.003	0.008 ± 0.001	ND

ND, not detectable; Data are shown as mean of three independent experiments (n = 3) ± SE

### Characterization of the substrate specificity of full active human CPD by quantitative peptidomics

**Fig 4:**
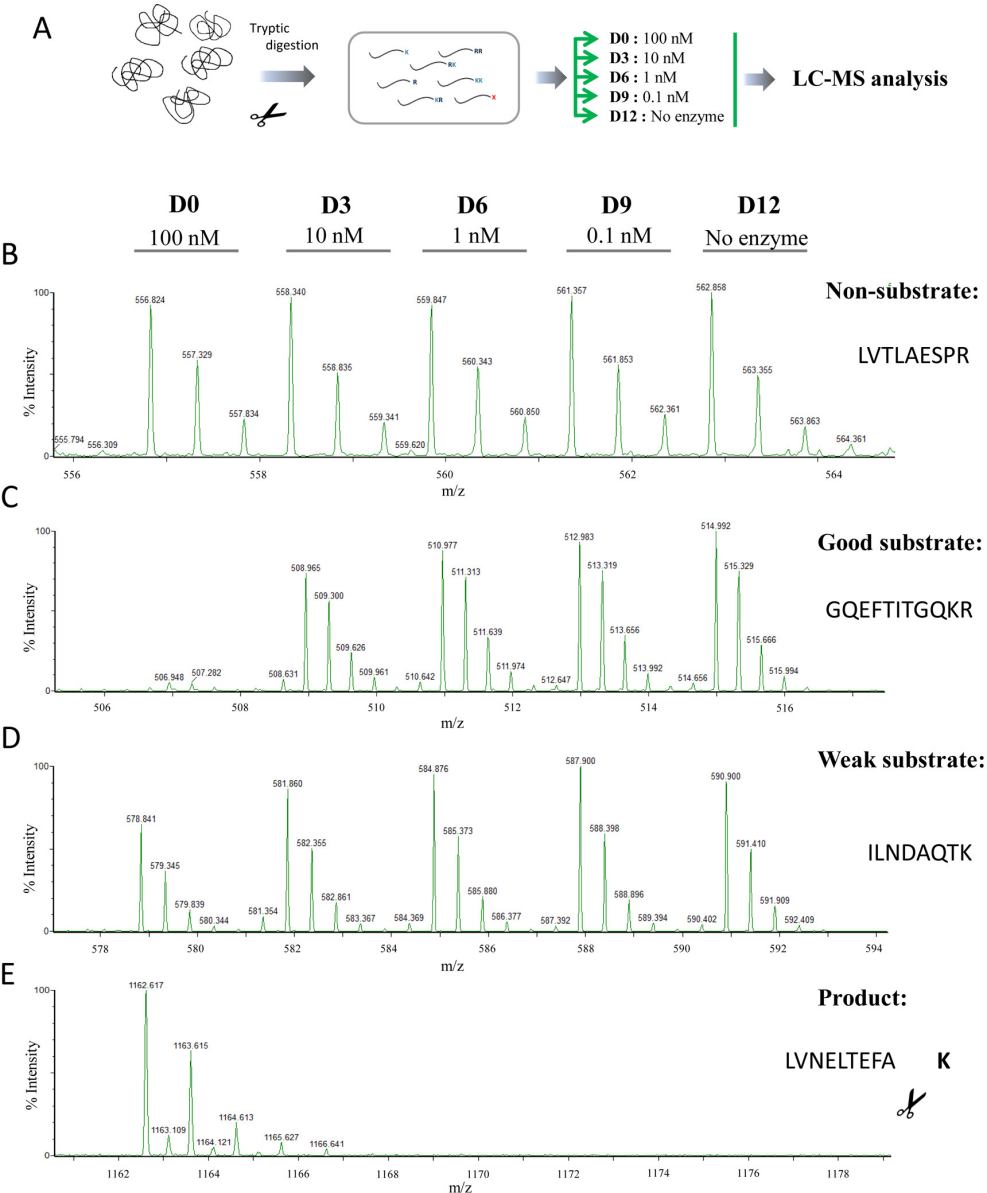
Quantitative peptidomics scheme for the characterization of rhCPD substrate specificity using the tryptic peptide library and representative spectra. (A) Quantitative peptidomics scheme. Tryptic peptides were obtained from digestion of five selected proteins (BSA, bovine thyroglobulin, bovine α-lactalbumin and human α and β–hemoglobin) with trypsin. The resultant peptide library was aliquoted and digested with no enzyme or different rhCPD concentrations of 0.1, 1, 10, and 100 nM for 16 h at 37°C. After incubation samples were labeled with one of five stable isotopic TMAB-NHS tags (D0 = 100 nM; D3 = 10 nM; D6 = 1 nM; D9 = 0.1 nM; D12 = No enzyme). Then, samples were pooled and analyzed by LC-MS. Examples of representative data are shown for (B) non-substrates, (C) good substrates, (D) weak substrates and (E) products.

The results obtained using synthetic substrates are useful, but is limited by the availability of substrates. To gain more information about the substrate specificity with a wide range of peptides, we used a quantitative peptidomic approach to address the substrate specificity of human CPD and unravel the contributions of each individual active domain. Quantitative peptidomics allows the study of proteolytic activity by analysing a large number of peptide substrates in a single experiment, thereby providing a more complete understanding of MCP substrate specificities [34,35,47,48]. First, to characterize the substrate specificity of the full active protein, we performed a peptidomics experiment in which different amounts of purified rhCPD (ranging from 0 to 100 nM) were incubated with a peptide mixture obtained by digestion of selected proteins with trypsin (see Materials and Methods section for more details). This tryptic peptide library contains mainly peptides with basic residues (Arg or Lys) on their C-termini, and only a small number of peptides lacking C-terminal basic residues which arose from either the C-terminus of the proteins or from non-tryptic cleavages. After incubation of aliquots of the peptide library with the indicated amount of CPD, the individual reactions were each labeled with a different isotopic TMAB tag, combined and analyzed by liquid chromatography/mass spectrometry (LC-MS) (see experimental scheme and representative data in **Fig 4**).

Over 55 peptides were identified by tandem mass spectrometry (MS/MS) and/or close matches with the theoretical mass of the peptides generated with trypsin. Some of these peptides exhibited a peak set with roughly equal peak heights, revealing that these peptides were not substrates or products of rhCPD under the experimental conditions used (**Fig 4B** and **S1 Table**). Some peptides were extensively cleaved, showing a complete or almost complete decrease in the peak intensity (≥60%) in the sample incubated with the highest concentration of enzyme (*i*.*e*., 100 nM) and a partial decrease in the peak intensity of the sample incubated with a lower concentration of rhCPD (*i*.*e*., 10 nM); these are considered as good substrates of rhCPD (**Fig 4C** and **Table 2**). In addition, some peptides were only partially cleaved (decrease in the peak intensity ≥20% and <60%), exhibiting a small decrease in intensity with the highest concentration of enzyme assayed, and no major decrease in the peak intensity with the concentration of 10 nM; these are considered as weak substrates of rhCPD (**Fig 4D** and **S2 Table**). Some peptides showed an increase in peak intensities that correlated with the amount of rhCPD. In all cases, the peptides that increased upon incubation with CPD lacked C-terminal basic amino acids and are products of rhCPD cleavage (**Fig 4E** and **S3 Table**). Based on the location of the peptide within the protein and the downstream sequence, we deduced the residue that was cleaved (see cleaved aa in **S3 Table**).

**Table 2:** Good substrates of rhCPD identified using the tryptic peptide library.

Protein precursor	Peptide sequence	Z	T	Obs M	Theor M	ppm	Ratio of peak intensity			
							rhCPD / No enzyme			
							100 nM	10 nM	1 nM	0.1 nM
Thyroglobulin	QQAAALAK	2	2	799.46	799.46	-4	<0.10	0.68	0.95	1.08
Thyroglobulin	FPLGESFLAAK	2	2	1178.64	1178.63	12	<0.10	0.94	1.12	1.09
α-Hemoglobin	VDPVNFK	2	2	817.43	817.43	2	<0.10	0.87	1.03	1.06
Thyroglobulin	GQEFTITGQKR	3	2	1263.67	1263.66	11	<0.10	0.75	0.96	0.95
Bovine serum albumin	ADLAK	2	2	516.28	516.29	-14	0.11	0.83	1.0	1.11
Thyroglobulin	KFEK	2	3	550.29	550.31	-38	0.11	0.67	0.85	1.07
Thyroglobulin	SLSLK	2	2	546.33	546.34	-15	0.13	0.89	0.98	1.13
Thyroglobulin	LPESK	2	2	572.32	572.32	-8	0.14	0.64	0.49	1.07
Thyroglobulin	KFEKLPESK	2	4	1104.61	1104.62	-5	0.15	0.66	0.96	1.19
Thyroglobulin	LTDEELAFPPLSPSR	2	1	1670.88	1670.85	20	0.16	0.78	1.10	1.07
Bovine serum albumin	LVNELTEFAK	2	2	1162.63	1162.62	12	0.20	0.94	1.09	1.09
α-Hemoglobin	LRVDPVNFK	3	2	1086.63	1086.62	13	0.21	0.88	1.09	1.06
Bovine serum albumin	LVTDLTK	2	2	788.47	788.46	9	0.40	0.84	1.05	1.08

Good substrates, peptides affected with a decrease ≥60% by the highest concentration of enzyme. Z, charge; T, number of isotopic tags incorporated into each peptide; Obs M, observed monoisotopic mass; Theor M, theoretical monoisotopic mass; ppm, difference between Obs M and Theor M (in parts per million); Peak intensity ratio rhCPD/no enzyme, the ratio in peak intensity between the sample incubated with enzyme and the sample incubated without enzyme. 100 nM, 10 nM, 1 nM and 0.1 nM refer to rhCPD concentration; When the peak intensity was below the background, the ratio is expressed as <0.10.

**Fig 5:**
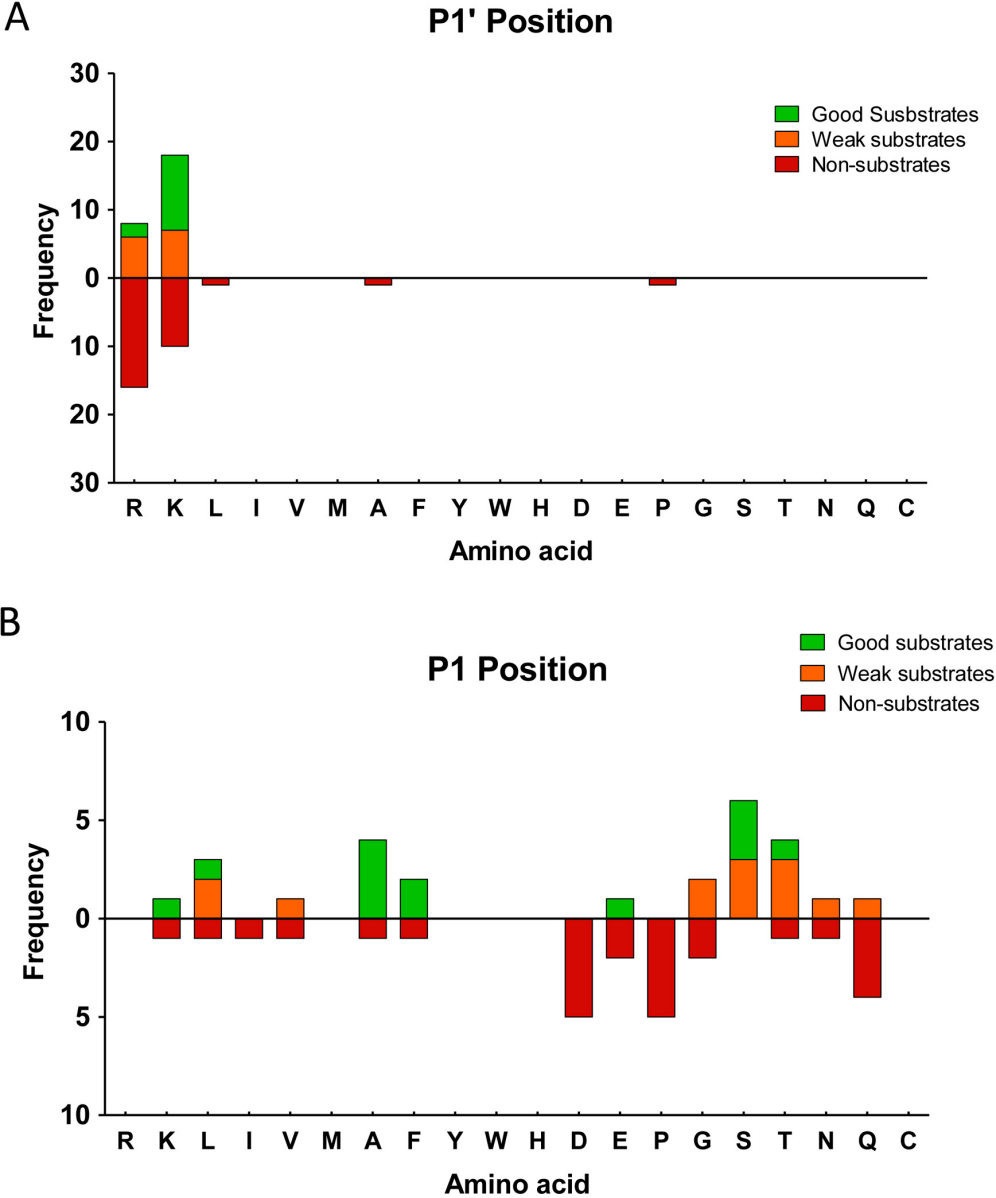
Analysis of the substrate preferences of rhCPD using the tryptic peptide library. (A) Substrate preferences of rhCPD at C-terminal (P1’) and (B) P1 positions. The number of times each amino acid was present in P1 or P1’ was determined for good substrates, weak substrates and non-substrates and represented. For P1 analysis, only substrates with permissive P1’ residues (Lys or Arg) were considered.

The majority of peptides with C-terminal (P1’) Lys were either good or weak substrates of rhCPD, while fewer of the peptides with C-terminal Arg were good substrates and the majority of these peptides were non-substrates (**Fig 5A** and **S1 Table**). Further, the influence of the penultimate (P1) residue on rhCPD substrates and non-substrates was examined (**Fig 5B**). For this analysis, only peptides with permissive C-terminal residues (Lys or Arg) were considered. Good substrates of rhCPD contained Ala, Ser, Phe, Leu, Lys, Thr, or Glu in the P1 position **(Fig 5B** and **Table 2)**. Weak substrates contained C-terminal Thr, Ser, Leu, Gly, Val, Glu, Asn or Gln (**S2 Table**). From these analyses, three products of rhCPD were detected; these peptides were generated by cleavage of peptides containing Arg or Lys at the C-termini and Ala, Phe or Thr in the P1 position (**S3 Table**). Finally, it is noteworthy to mention that no peptide with Pro, Asp, or Ile in the P1 position ws identified as a rhCPD substrate. Some of these peptides contained C-terminal basic residues (including Arg and Lys), which suggest an important negative influence of these residues on the CPD activity when they are found in P1 position.

During the peptidomic analysis, peptides containing Cys, Tyr, or His showed variable peak intensities that did not correlate with the amount of CPD. In addition, peptides containing cyanoCys, mono-iodoTyr, di-iodoTyr, mono-iodoHis, and di-iodoHis were identified from MS/MS analysis of the data; these peptides result from chemical reactions due to impurities in the TMAB labels [49]. This finding might explain the absence and/or relatively low abundance of peptides with Cys, Tyr, or His residues in some previous peptidomics studies which used TMAB tags [34,35,47–50]. Consequently, peptides with Tyr, His, or Cys were not considered for the present work.

### Characterization of the substrate specificity of human CPD domains I and II by quantitative peptidomics

**Fig 6:**
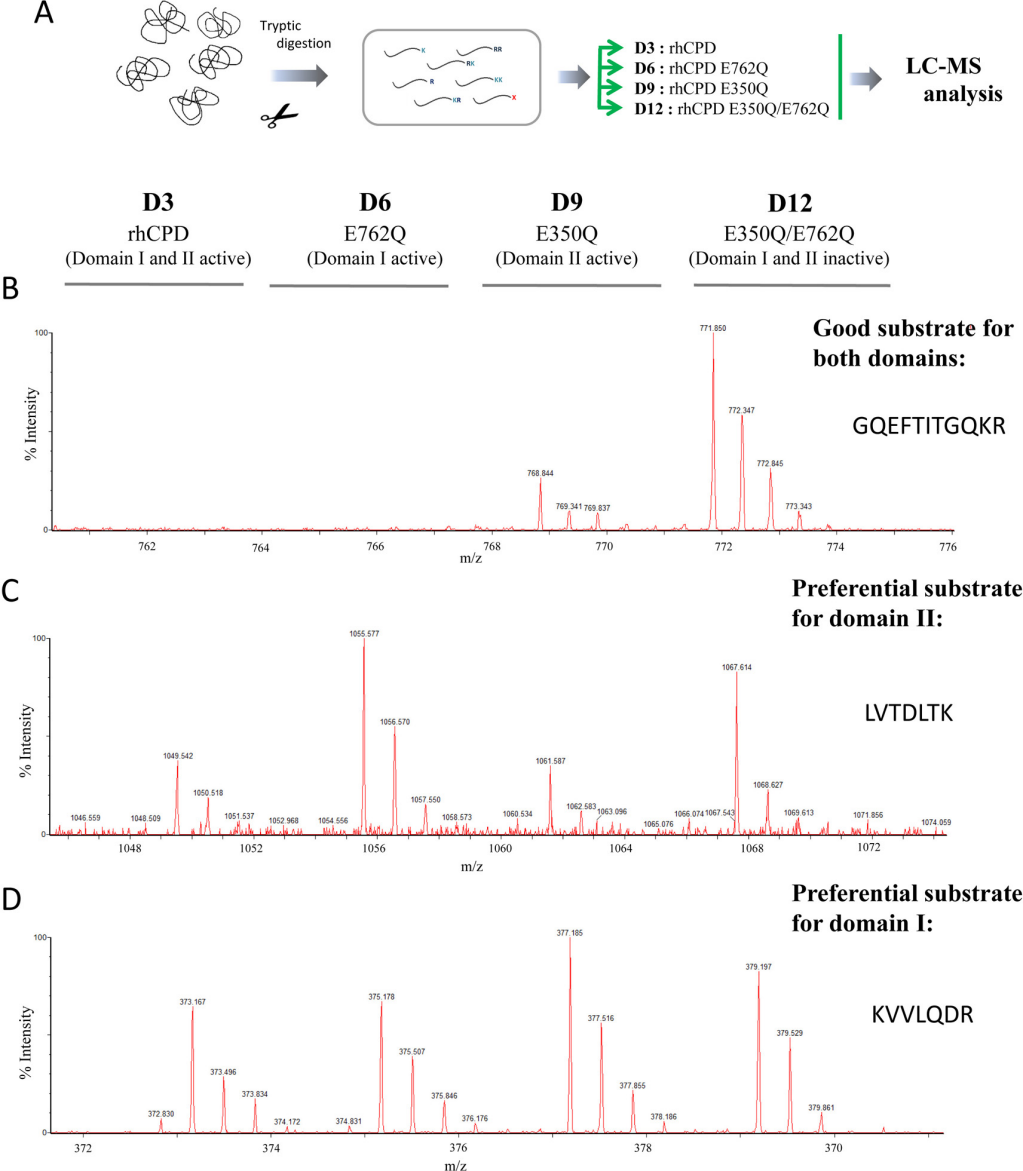
Quantitative peptidomics scheme for the substrate characterization of CPD domains I and II using a tryptic peptide library and representative spectra. (A) Quantitative peptidomics scheme. Tryptic peptides were obtained as described above. The resultant peptide library was aliquoted and digested with 100 nM rhCPD, 100 nM rhCPD E350Q, 100 nM rhCPD E762Q or 100 nM rhCPD E350Q/E762Q for 16 h at 37°C. Then, samples were labeled with one of the isotopic TMAB-NHS tags. For the representative data shown in this figure, D3 = rhCPD; D6 = rhCPD E762Q; D9 = rhCPD E350Q; and D12 = rhCPD E350Q/E762Q. Finally, samples were pooled and analyzed by LC-MS. Examples of representative data are shown for (B) good substrates of both domains I and II, (C) preferential substrates of domain II and (D) preferential substrates of domain I. The entire experiment was performed three times and the various enzyme digests were labelled with different tags to control for variability of the individual reagents.

To gain insight into the substrate specificity of the domains I and II of human CPD, we repeated the quantitative peptidomics and compared rhCPD with the various mutant forms in a single LC/MS run. A schematic of one experiment and labeling strategy is shown in **Fig 6A**; we performed at least three biological replicates of each enzyme form. For this analysis, a single concentration (100 nM) of purified rhCPD, of single point mutants E350Q and E762Q, and the double mutant E350Q/E762Q were incubated with the same tryptic peptide library used for the characterization of rhCPD described above. After incubation with the enzyme, the individual reactions were differentially labeled with isotopic TMAB tags, combined and subjected to LC-MS analysis (**Fig 6A**).

Over 48 peptides were identified through tandem mass spectrometry (MS/MS) and/or by close matching with the theoretical mass of the peptides generated with trypsin. After the analysis, the results revealed some peptides which showed roughly equal peak heights; these peptides were considered to be neither substrates nor products under the assay conditions used (**S4 Table**). Some peptides were cleaved by the enzyme with both domains I and II active, showing a complete or almost complete decrease in the peak intensity upon incubation with rhCPD; these are considered as substrates (which include good and weak substrates) of rhCPD (**Fig 6B** and **Table 3**). Among them, some peptides showed a complete or almost complete decrease in the peak intensity after incubation with either single point mutant E350Q or E762Q (**Fig 6B** and **Table 3**); these are considered as substrates of both domains. However, some peptides substrates of rhCPD revealed a complete or almost complete decrease in the peak intensity upon incubation with the single point mutant E350Q and a slight or no decrease in the peak intensity upon incubation with E762Q; these are considered as preferential substrates of domain II (**Fig 6C** and **Table 3**). Another group of peptides revealed a moderate decrease in the peak intensity upon incubation with the single point mutant E762Q and a slight or no decrease in the peak intensity upon incubation with the E350Q mutant; these are considered as preferential substrates of domain I (**Fig 6D** and **Table 3**).

**Table 3:** Good and weak substrates identified within substrate characterization of CPD domains I and II using the tryptic peptide library.

Protein precursor	Peptide sequence	Z	T	Obs M	Theor M	Ppm	Ratio of peak intensity enzyme / Control			Ratio of peak intensity dI active / d II active
							rhCPD	Domain I active	Domain II active	
Thyroglobulin	GQEFTITGQKR	2	2	1263.66	1263.66	4	<0.10	<0.10	0.16	<0.10
α-Hemoglobin	VDPVNFK	2	2	817.42	817.43	-20	<0.10	1.05	<0.10	>5.00
Thyroglobulin	FAATSFR	2	1	798.40	798.40	-3	0.12	<0.10	0.24	<0.10
Bovine serum albumin	LVNELTEFAK	2	2	1162.62	1162.62	1	0.27	0.65	<0.10	>5.00
Bovine serum albumin	LVTDLTK	2	2	788.45	788.46	-16	0.33	0.83	0.29	3.53
α-Hemoglobin	LRVDPVNFK	3	2	1086.57	1086.62	-44	0.34	1.09	<0.10	>5.00
Bovine serum albumin	KQTALVELLK	3	3	1141.66	1141.71	-41	0.42	1.09	0.42	2.60
Bovine serum albumin	AEFVEVTK	2	2	921.48	921.48	0	0.43	0.75	0.41	1.84
Thyroglobulin	KVVLQDR	2	2	856.49	856.51	-18	0.54	0.52	0.66	0.86
Thyroglobulin	AFLGTVR	2	1	762.44	762.44	5	0.59	0.56	0.53	1.07
Bovine serum albumin	KVPQVSTPTLVEVSR	3	2	1639.00	1638.93	41	0.59	0.96	0.51	1.86
Thyroglobulin	VVLQDR	2	1	728.42	728.42	0	0.64	0.79	0.79	1.00
Thyroglobulin	AVKQFEESQGR	3	2	1277.64	1277.64	3	0.64	0.75	0.75	1.00
Thyroglobulin	AISVPEDIAR	2	1	1069.63	1069.58	46	0.65	0.69	0.59	1.18
Thyroglobulin	ASGLGAAAGQR	2	1	957.52	957.50	17	0.72	0.56	0.83	0.67
Thyroglobulin	GQEIPGTR	2	1	856.47	856.44	33	0.72	0.59	0.59	1.00
Bovine serum albumin	IETMR	2	1	648.31	648.33	-26	0.74	0.59	0.74	0.80
Thyroglobulin	ELSVLLPNR	2	1	1039.64	1039.60	43	0.74	0.78	0.78	1.00
Thyroglobulin	KGQEFTITGQK	3	3	1235.63	1235.65	-15	0.78	0.99	0.74	1.33

Good substrates, peptides affected with a decrease ≥60% by rhCPD. Weak substrates, peptides affected with a decrease ≥20% and <60% by rhCPD. Ratio of peak intensity enzyme/control, the ratio in peak intensity between the sample incubated with rhCPD, E762Q or E350Q (as indicated for rhCPD, domain I active or domain II active, respectively) and the sample incubated with the double mutant (E350Q/E762Q). Ratio of peak intensity d I active /d II active, the ratio in peak intensity between the sample incubated with E762Q and the sample incubated with E350Q. See Table 2 for the rest of abbreviation definitions.

**Fig 7:**
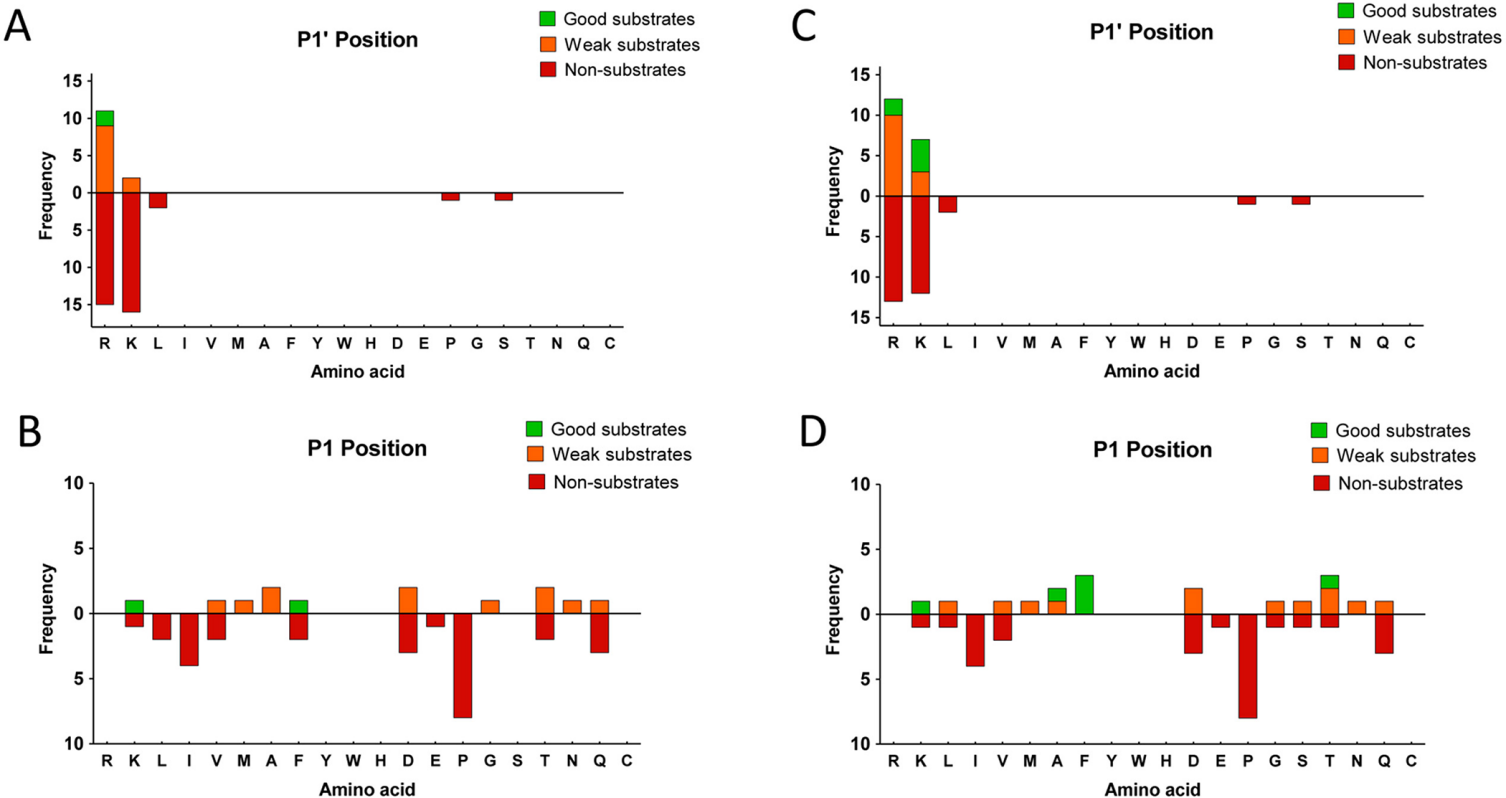
Analysis of the substrate preferences of CPD domains I and II using a tryptic peptide library. (A,B) Substrate preferences of CPD domain I (*i*.*e*., rhCPD E762Q). (A) C-terminal P1’ position preferences. (B) P1 position preferences. (C,D) Substrate preferences of CPD domain II (*i*.*e*., rhCPD E350Q). (C) C-terminal P1’ position preferences. (D) P1 position preferences. The number of times each amino acid was present in P1 or P1’ was determined for good substrates, weak substrates and non-substrates. For P1 analysis, only substrates with permissive P1’ residues (Lys, Arg) were considered.

Analysis of the P1’ residue of substrates of domain I showed a predominance of Arg, with 11 substrates containing C-terminal Arg and only 2 substrates containing C-terminal Lys (**Fig 7A**). In contrast, substrates of domain II were more evenly divided between C-terminal Lys versus Arg (**Fig 7C**). Peptides with C-terminal Arg and Lys were also identified as non-substrates of the mutant CPD with single catalytic domains. For these peptides, the identity of the penultimate (P1) residue was further analyzed (**Fig 7B and 7D**). Good substrates of the CPD domain I contained only Lys or Phe amino acids in this position. Weak substrates of CPD domain I contained Ala, Thr, Asp, Val, Met, Asn, Gln or Gly in the P1 position **(Fig 7B)**. Good substrates of the CPD domain II contained Phe, Lys, Ala or Thr in the P1 position. Weak substrates of the CPD domain II contained Asp, Thr, Ala, Leu, Ser, Met, Val, Asn, Gln or Gly in this position **(Fig 7D)**. Again, no peptides with Pro, Ile, or Glu in the P1 position were identified as substrates of either domain under the experimental conditions performed.

Six peptides were identified as products of rhCPD and the domain II only active mutant; these resulted from cleavage of Arg or Lys from tryptic peptides, with Ser, Phe or Val at the P1 position of the cleavage site (**S5 Table**). Three of these peptides required removal of C-terminal Arg, and these were also products of domain I. The other three peptides required removal of C-terminal Lys, and two of these peptides were only products of domain II and not domain I. The peptide LRVDPVNF was greatly elevated upon incubation with CPD with active domain II, and slightly elevated by incubation with CPD with active domain I (**S5 Table**). Because the corresponding tryptic peptide contains a Lys on the C-terminus, this finding shows that CPD domain I is capable of cleaving C-terminal Lys, although not as efficiently as CPD domain II cleaves C-terminal Lys from this peptide.

### Human CPD cleaves exclusively C-terminal basic residues

**Fig 8:**
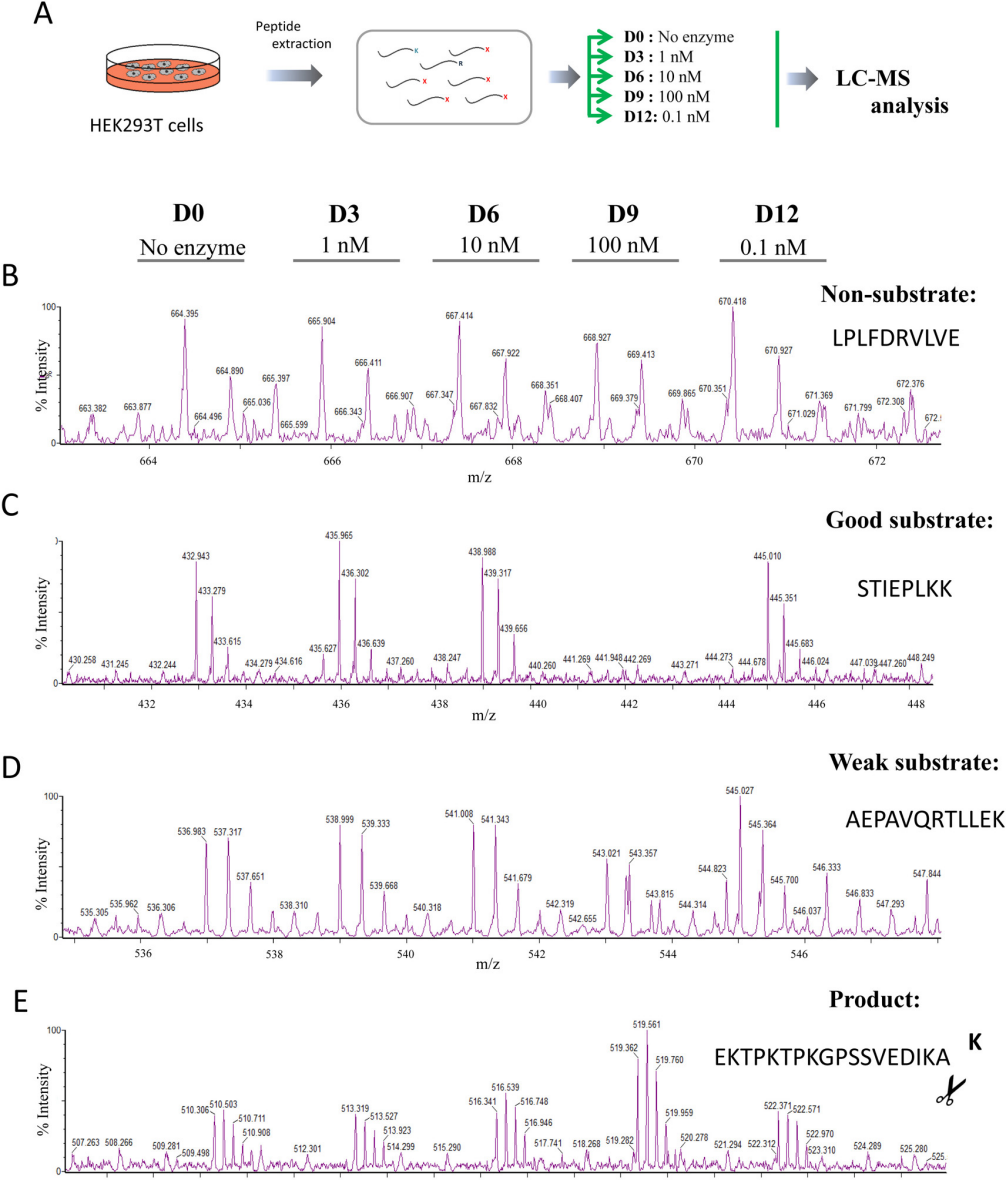
Quantitative peptidomics scheme for the study of rhCPD substrate specificity using HEK293T derived peptide library and representative spectra. (A) Quantitative peptidomics scheme. Peptides were extracted from HEK293T cell cultures treated for 1 h at 37°C with 0.5 μM bortezomib. The resultant peptide extract (*i*.*e*., HEK293T peptidome) was aliquoted and digested with no enzyme or different rhCPD concentrations *(i*.*e*., 0.1, 1, 10, and 100 nM at 37°C for 16 h). After incubation samples were labeled with one of five stable isotopic TMAB-NHS tags (D0 = No enzyme; D3 = 1 nM; D6 = 10 nM; D9 = 100 nM; and D12 = 0.1 nM). Then, samples were pooled and analyzed by LC-MS. Examples of representative data are shown for (B) non-substrates, (C) good substrates, (D) weak substrates and (E) products.

The tryptic peptide library provided a large number of peptides with C-terminal basic residues, but few peptides without C-terminal basic residues. To determine if rhCPD was able to cleave any peptide lacking C-terminal basic residues, we performed a third quantitative peptidomics study testing peptides extracted from HEK293T cells treated with bortezomib, a proteasome inhibitor that causes an increase in the levels of many intracellular peptides [51]. After incubation of the peptides with CPD, the individual reactions were differentially labeled with isotopic TMAB tags, combined and analyzed by LC-MS (see experimental scheme in **Fig 8A**).

**Fig 9:**
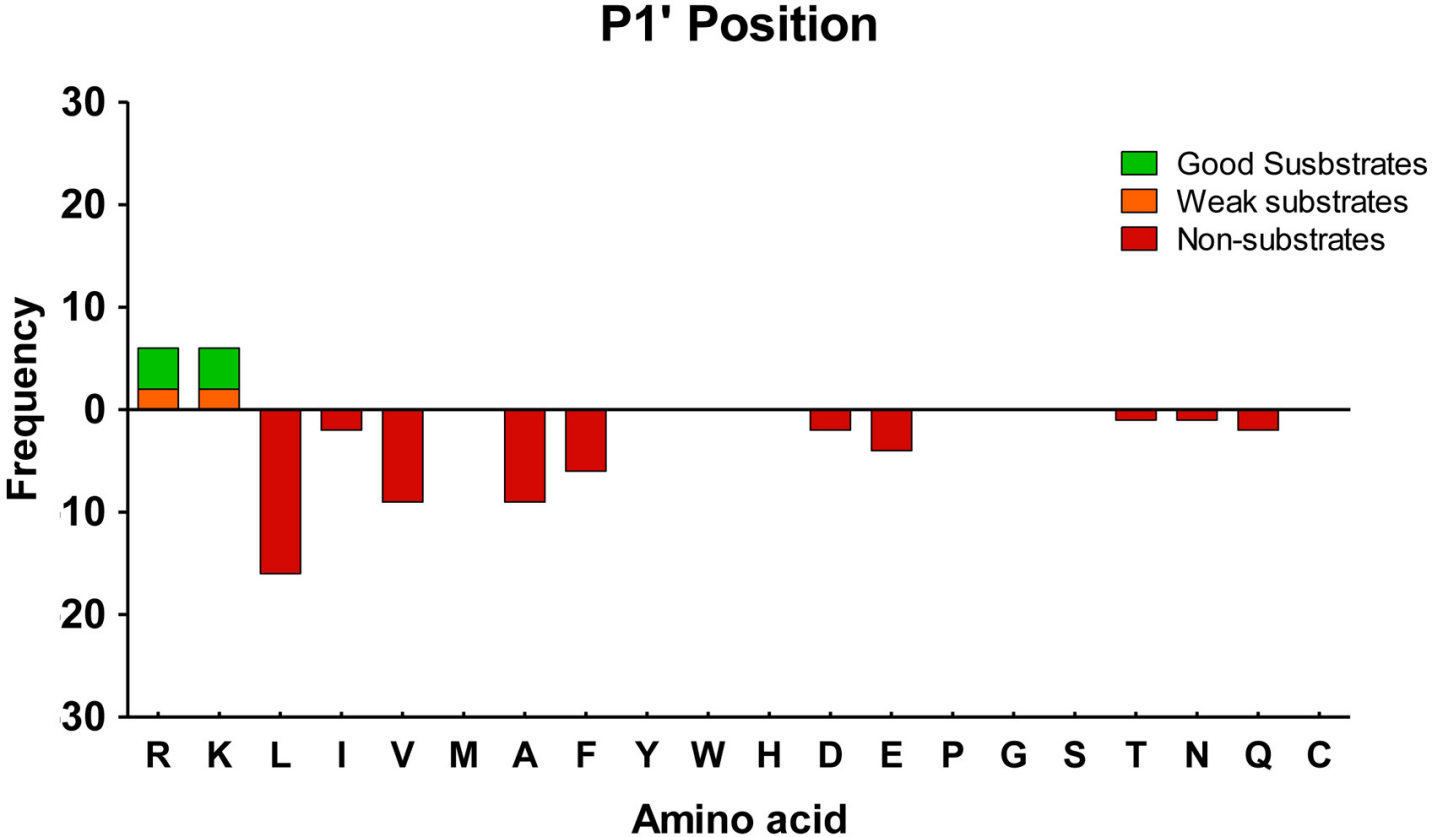
Analysis of the P1’ substrate preferences of rhCPD using a HEK293T derived peptidomics library. The number of times each amino acid was present in P1’ was determined for good substrates, weak substrates and non-substrates.

More than 60 peptides were identified through tandem mass spectrometry (MS/MS). Unlike the tryptic peptide library, in the HEK293T derived library the majority of peptides identified did not contain C-terminal basic residues. Nevertheless, none of these non-basic ending peptides was affected by the treatment with rhCPD, and thus exhibited a peak set with roughly equal peak heights (**Fig 8B and S6 Table**). Of the peptides that contained C-terminal basic residues, some were extensively cleaved, showing a complete or almost complete decrease in the peak intensity upon incubation with the highest concentration of enzyme of 100 nM and a partial decrease in the peak intensity with a lower concentration of 10 nM; these are considered as good substrates of rhCPD (**Fig 8C** and **Table 4**). Some peptides were only partially cleaved, exhibiting a small decrease in intensity with the highest concentration of enzyme assayed and no or slight decrease in the peak intensity with the concentration of 10 nM; these are considered as weak substrates of rhCPD (**Fig 8D** and **S7 Table**). A small number of peptides showed an increase in the peak intensities related with increasing amounts of rhCPD; these are considered as products resultant after the rhCPD cleavage (**Fig 8D** and **S8 Table**). As expected, analysis of the P1’ residue showed again an exclusive preference for basic residues at this position (**Fig 9**).

**Table 4:** Good substrates of rhCPD identified using HEK293T peptides.

Precursor	Sequence	Z	T	Obs M	Theor M	ppm	Ratio of peak intensity			
							rhCPD / No enzyme			
							100 nM	10 nM	1 nM	0.1 nM
Eukaryotic translation initiation factor 5A	SAMTEEAAVAIKAMAK	3	3	1620.84	1620.821	11	<0.10	0.98	1.02	0.90
Acidic nuclear phosphoprotein pp32	STIEPLKK	3	3	914.53	914.54	-20	<0.10	1.12	1.15	1.00
40S Ribosomal protein S21	KADGIVSK	3	3	816.45	816.470	-20	<0.10	0.83	0.89	0.96
Histidine triad nucleotide-binding protein 1	Ac-ADEIAKAQVAR	2	1	1212.67	1212.646	21	0.15	1.03	1.03	1.14
Vimentin	AELEQLKGQGKSR	3	3	1442.78	1442.78	0	0.23	0.95	0.97	1.11
Eukaryotic translation initiation factor 5A	SAMoxTEEAAVAIKAMAK	3	3	1636.83	1636.821	3	0.32	0.98	1.00	0.94
Eukaryotic translation initiation factor 5A	NMDVPNIKR	3	2	1085.57	1085.565	4	0.38	0.95	1.05	0.72
Hematological and neurol. exp. 1 prot.	Ac-TTTTTFKGVDPNSRNSSR	3	1	2010.02	2009.977	20	0.40	1.13	1.07	0.97

See Tables 2 and 3 for abbreviation definitions

### Comparative modeling of the active sites of human CPD domains I, II and III

To date, a number of crystallographic studies have delimited the active site residues that play a role in the catalytic mechanism of the M14B subfamily of MCPs [43,44,52–54]. As the three-dimensional structures of human CPD domains are not yet available, we modeled all three human domains based on the structures of duck and *Drosophila melanogaster* CPD domains previously solved by our groups [43,44]. These models allow for the analysis of the structural elements that contribute to CPD substrate preferences and biological activity.

**Fig 10:**
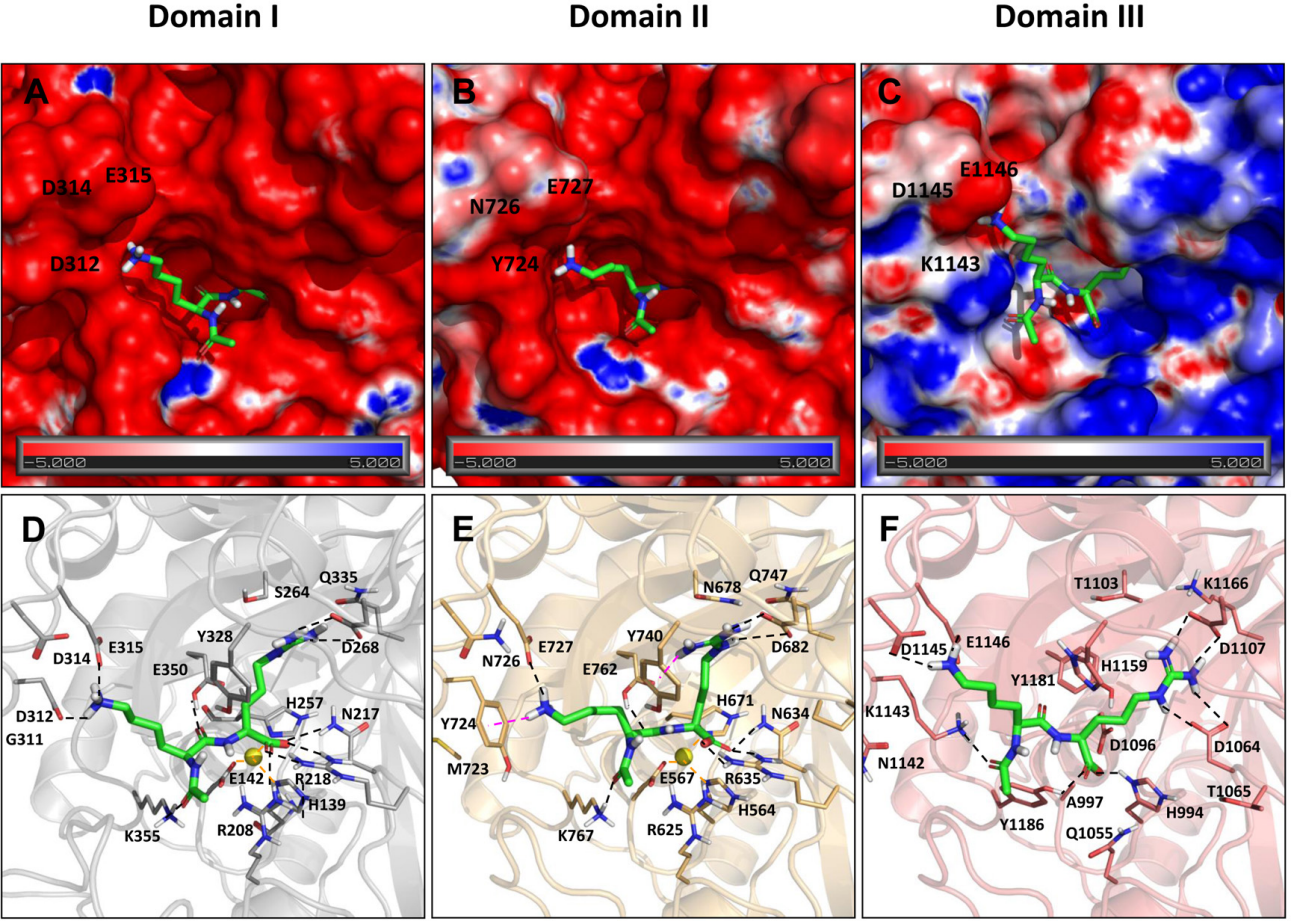
Structural modeling of the active sites of CPD domains I, II and III. Electrostatic potential molecular surfaces of the catalytic sites of CPD domains I (A), II (B) and III (C) in presence of a modeled peptide. Lower panels show the best docking pose based on GlideScore for the truncated peptide GQKR (green sticks) within the active sites of domains I (D), II (E), and III (F), with the two last N-terminal residues omitted for clarity. Zn coordinates (yellow sphere) are added from the template structure used for modeling (PDB 1H8L). Hydrogen bonds are depicted in black dashed lines, π-cation interactions in pink and metal coordination in orange dashed lines. Within lower panels, the side chains of residues directly involved in the zinc binding are shown (i.e., His69, Glu72 and His196 using bCPA1 numbering; corresponding to His139/564, Glu142/567, and His257/671 in CPD domains I/ II), as well as the catalytic residue Glu270 (Glu350/762 in CPD domains I/II). In addition, the side chains of some of the putative residues determining respectively the S1’ and S1 specificity pockets are shown for domains I/ I; *i*.*e*., Arg218/635 (corresponding to Arg145 of bCPA1 numbering); Tyr328/740 (Tyr248 of bCPA1); and Gln335/747 (Ile255 of bCPA1). See S3 Fig for other residue equivalences.

The derived models of human CPD domains display active site clefts that are located at the edge of the active-sites grooves within the β-sheet of the catalytic moiety, in agreement with other MCP structures. Inside the catalytic cleft is found the typical catalytic zinc metal ion, which is coordinated with one Glu and to two His residues. The basis of the active-site groove is formed by strands β5, β6, β8 and the N-terminal part of helix α2. The wall of the groove funnel is essentially defined by two projections of the α4–α5 multiple turn and by the α7–β7 hairpin loop, as described previously [43,44,52–54]. **Fig 10** (panels A-C) shows the electrostatic potential molecular surfaces of the catalytic sites of CPD domains I, II and III, highlighting the differences in electrostatic properties for the three domains. While domain I and II are more similar in terms of electrostatic potential, the active site of domain III completely differs in the distribution of its electrostatic surface potential. In addition, a characterization of the acceptor maps (**S2 Fig**, panels A-C) and donor maps (**S2 Fig**, panels D-F) for these three domains shows that they differ mainly in the properties of the S1’ and S1 region. Domain I has a more clear preference for donor groups in S1 region, due to the acidic properties of some of the residues involved in this site (Asp312, Asp314 and Glu315), as compared with domain II (equivalent residues in S1 region are Tyr724, Asn726 and Glu727). This observation is also reflected in the electrostatic potential maps.

As shown in the multiple sequence alignment in **S3 Fig**, both domains I and II conserve the three protein ligands of the catalytic zinc ion (His69, Glu72 and His196, based on the conventional numbering system for the active form of bovine CPA1) and the residues directly involved in catalysis (Arg127, Arg145 and Glu270, according to the bovine CPA1 numbering). Further, CPD domains I and II display the same residues involved in the S1’ substrate binding subsite (*i*.*e*., Asp and Gln residues in equivalent positions to 207 and 255 in bovine CPA1, respectively), determining a substrate specificity for basic residues. Nonetheless, human CPD domains I and II differ in some residues within the S1’ specificity pocket. One of the major differences is that human CPD domain I contains Ser264 in a position comparable to Leu203 of bovine CPA1 and Ser198 of CPM (**S3 Fig**), whereas CPD domain II contains Asn678 in an equivalent position. Relevant residues at the S1 subsite, such as Tyr248 and Ser199 (following the bovine CPA1 numeration) are essentially conserved between both CPD domains I and II, whereas notable residue variations are observed in one of the loops (α7–β7 hairpin loop) that shapes the border of the funnel and S1 subsite (see **S3 Fig**). The equivalent loop in CPM structure has been proposed to play an important role to accommodate the long hydrophobic P1 side chain of bradykinin [52].

Unlike domains I and II, human CPD domain III lacks the majority of key residues for CP catalysis (**S3 Fig**). In this domain, only the first His residue involved in zinc binding (*i*.*e*., His69, according to the bovine CPA1 numeration) is present; in contrast, the other two residues involved in metal binding (*i*.*e*., Glu72 and His196, following bovine CPA1 numeration) are replaced by Ala and Asp residues, a change conserved in duck CPD domain III [54]. While Asp can coordinate zinc, Ala is not able to perform such function. Further, in the human domain III, two residues involved in the catalytic mechanism (*i*.*e*., Glu270 and Arg127, according to bovine CPA1 numeration) are occupied by Tyr and Gln, respectively. Residues Asn144, Arg145 and Asn146 in bovine CPA1, responsible for the anchoring of the carboxylate group of the substrate, are replaced by a triad of Asp, Thr and Asp in human CPD domain III. Interestingly, the enzyme γ-D-glutamyl-(L)-meso-diaminopimelate peptidase I, which is a member of the M14B subfamily of MCP and a distant relative of CPD, has a triad of Asn, Asp and Gln in homologous positions to the triad Asn144/Arg145/Asn146. Such residue replacement confers to this bacterial enzyme a particular substrate specificity for C-terminal meso-diaminopimelic acid [6,55].

In order to understand the detailed interactions between the peptides substrates within the catalytic sites of the different CPD domains, we performed a preliminary docking experiment with the GQEFTITGQKR peptide, which shows the highest preference for both active domains (See **Table 2 and Table 3**). For the sake of simplicity, we used a truncated version of the peptide; *i*.*e*., the tetrapeptide GQKR. The best docking pose for GQKR in domain I shows that the C-terminal residue, arginine, interacts with the S1’ region formed by Asp268, Gln335 and Ser264 **(Fig 10A and 10D and S4 Fig**). As expected from homologous systems, the terminal carboxylate group interacts with the substrate-binding residues Arg218 and Asn217, while the peptide bond subjected to cleavage is located near Arg208. The Zn ion and the main catalytic residue, Glu350, are also surrounding this peptide bond. On the other hand, the lysine at P1 position, places the side chain in the previously described S1 region, interacting with the acidic residues Asp312, Asp314 and Glu315 located at the α7–β7 hairpin loop (**Fig 10A**). In fact, the S1 region displays a marked negative surface electrostatic potential that may explain its preference for basic residues in P1 position. In the case of domain II, GQKR binding mode shows similar interactions for the S1’ region, establishing contacts with Asp682 and Gln747 (**Fig 10B and 10E and S4 Fig**). Residues Arg635 and Asn634 are stabilizing the C-terminal group, while Arg625, Zn and Glu762 interact with the peptide bond. The lysine at the P1 position in the peptide also interacts with the S1 region, but the type of interactions differ from those of domain I. The positive charge of the lysine makes a π-cation interaction with Tyr724 as well as hydrogen bond contacts with Glu727. In summary, although the binding mode of the tetrapeptide is well reproduced in the two domains, differences in regions S1 and S1’ may explain the differences in substrate specificity.

As previously explained, the active site of domain III lacks the main catalytic residues, thus being unable to cleave conventional CP substrates. As the function of this domain remains unknown, we wanted to assess its ability to accommodate peptides in its active site. Docking studies with the truncated peptide GQRK predict that this domain is able to bind the peptide with a similar binding mode (**Fig 10C and 10F**). The arginine in the C-terminal end interacts strongly with Asp1107, which corresponds to Asp268 and Asp682 in domains I and II, respectively. There is an additional interaction with Asp1064. Lysine in position P1 interacts with Asp1145 and Glu1146, the latter being conserved in the three domains. However, there are some differences in the rest of the residues that form the S1 region. In particular, Asp312 from domain I is aligned to Tyr724 in domain II and Lys1143 in domain III. The properties of these three residues are different enough and may explain the different affinity of the domains for substrates. Accordingly, these results support the hypothesis that domain III might be able to bind peptides and may perform a non-enzymatic function, such as peptide binding and/or transport.

### Molecular docking can help to explain substrate specificity of human CPD domains I and II

In order to fully explain the different substrate specificities for the two active CPD domains, we decided to perform an extensive molecular docking study. For this purpose, we selected six peptides, based on the different experimental ratio values observed (i.e. including good, weak and non-substrates for both domains I and II) shown in **Table 3** and **S9 Table**. The longest peptides were truncated to 7 C-terminal residues in order to simplify the docking calculations. The final set of peptides included TITGQKR, LVTDLTK, GAAAGQR, FAATSFR, VDPVNFK and SALGEPKK. The analysis of the best docking poses for these peptides did not explain the different substrate specificity of the two domains, in terms of docking score or particular peptide/receptor interactions. For instance, the best docking poses obtained for VDPVNFK and FAATSFR peptides show very similar interactions, which does not explain why these two peptides have different preference towards the CPD domain I. Therefore, we performed an additional analysis of the docking results, based on the number of expectedly correct docking solutions found within all resulting docking poses for each peptide. The criteria to consider a docking peptide pose as correct are based on the following features: the center of mass of the P1’ and P1 residues should be within 10 Å distance from the center of mass of CPD domain I Asp268 (S1’ pocket) and Glu315 (S1 pocket) residues, respectively, or from CPD domain II Asp682 (S1’ pocket) and Glu727 (S1 pocket) residues, respectively. These criteria are likely to describe the expected general interactions in peptide binding as discussed in the previous section. From this analysis of the docking results, we found that the percentage of correct docking solutions (respect to 101 docking poses analyzed) for each peptide is a good indicator of the observed cleavage of that peptide by each domain; *i*.*e*., the higher the percentage of correct poses, the better performance as substrate in the experimental data **(S9 Table and S5 Fig).** For instance, a high percentage of correct docking poses for domain I was found in the case of TITGQKR and FAATSFR peptides. On the other hand, domain II showed preference to bind correctly TITGQKR, LVTDLTK, FAATSFR and VDPVNFK.

## Discussion

The primary goal of the present study was the detailed comparison of the substrate specificity of the first and second catalytic domains of CPD, as well as testing the third carboxypeptidase-like domain for catalytic activity using a broad array of peptides. CPD is the only member of the MCP family that contains multiple carboxypeptidase domains [11,15,16]. Proteolytic enzymes with multiple catalytic sites are rare in nature, especially enzymes with catalytic sites that perform generally similar roles such as domains I and II of CPD. Angiotensin-converting enzyme (ACE) is another peptidase with two active domains in most species and tissues (the form produced in sperm contains a single catalytic domain). The two domains of ACE have similar catalytic activities that differ mainly in their sensitivity to chloride and substrate preferences [56,57]. Another group of enzymes with multiple domains that perform related catalytic functions are the polyserases. As with CPD, the polyserases contain enzymatically active and inactive domains of unknown function. Polyserase-1 is post-translationally cleaved into distinct subunits that contain a single catalytic activity, but polyserase-2 and -3 remain intact as multi-domain-containing proteins [58–60]. The presence of multiple catalytically active domains can increase the diversity of substrates cleaved, or the efficiency of the enzyme under different cellular conditions. Both appear to be the case for the two active domains of CPD.

One important contribution of the present study is that human CPD domain I and II have distinct pH optima, with domain I working best at neutral pH while domain II is optimal at mildly acidic pH values. This observation is consistent with the properties previously reported for duck and *Drosophila* CPD [16,22]. indicating that this feature is highly conserved through hundreds of millions of years of evolution. Although CPD is primarily detected in the trans Golgi network, its presence there is not static. CPD is present in vesicles that bud from the trans Golgi, but is retrieved from these vesicles and sorted back to the trans Golgi. CPD is also transiently found on the cell surface, where it is internalized and a fraction is transported through the endocytic pathway back to the trans Golgi network [17]. The pH of each of these compartments is different, ranging from neutral (the cell surface) to slightly acidic (the trans Golgi network) to acidic (endosomes). The broad pH range of CPD domain II implies that this activity is functional in the various cellular compartments, while CPD domain I is optimally active on the cell surface where the pH is neutral.

Another important finding of the present study is that human CPD domain I and II have differences in their substrate specificities. Both are specific for C-terminal basic residues, with no detectable cleavage of peptides with non-basic residues on the C-terminus. The present study tested dozens of peptides in a peptidomic assay, and extended the previous studies performed with duck and *Drosophila* CPD which tested a small number of substrates [16,22]. The preference of CPD domain I for Arg over Lys, and the broad ability of domain II to cleave both Arg and Lys with comparable efficiency was previously noted in studies that tested duck and *Drosophila* CPD with a single pair of peptides that differed only in the C-terminal residue [16,22]. The present study tested more than 100 peptides, and while each domain was able to cleave either Lys or Arg from some peptides, there was a clear preference for domain I to cleave Arg and not Lys. Interestingly, despite this strict specificity towards basic residues of both domains, in our study we found some peptides containing this residue that were not cleaved by domain I or II of CPD. These peptides contained Pro, Asp or Ile in P1 position, confirming that other residues different from the P1’ affect the efficiency of this human enzyme. Taken together with the pH optima, it appears that when present on the cell surface, CPD domain I will be active and cleave peptides/proteins with C-terminal Arg, while CPD present in the trans Golgi and endocytic pathways will have domain II active and cleave both C-terminal Lys and Arg.

Previous studies using small numbers of substrates have found that other members of the M14B subfamily of MCPs are more efficient at cleaving C-terminal Arg than Lys. For example, CPM cleaves Met^5^-Arg^6^-enkephalin with a *k*_cat_ of 934 min^-1^ and *K*_m_ of 46 μM, whereas Met^5^-Lys^6^-enkephalin is cleaved with *k*_cat_ of 663 min^-1^ and *K*_m_ of 375 μM [61]. Other studies evaluated the specificity of MCPs using peptides with C-terminal Arg or Lys residues as competitive inhibitors. For example, CPE is inhibited by Leu^5^-Lys^6^-enkephalin with a k_i_ of 174 μM and by Leu^5^-Arg^6^-enkephalin with a k_i_ of 83 μM [62]. CPZ is inhibited by hippuryl-Arg but not by hippuryl-Lys [63]. Taken together with the present studies, CPZ and CPM are like CPD domain I with a strong preference for C-terminal Arg over Lys, while CPE and CPN are more like CPD domain II which does not have a marked preference for one basic residue over another. One residue within the substrate binding pocket that correlates with selectivity is the residue in position equivalent to Leu203 in CPA1. In CPD domain I Ser264 is present in this position, while in CPD domain II an Asn678 is present (**Fig 10**). Similarly, CPM and CPZ contain a Ser in this position, and CPE and CPN contain Asn. Perhaps the smaller side chain of the Ser (versus Asn) within the binding site has a superior capability to fit and facilitate the establishment of contacts between the guanidine group of the C-terminal scissile Arg residue, leading to major preference for Arg- versus Lys-containing substrates. The analysis of the docking landscape suggests that there might be some kinetic effects involved in the processes of approaching and entering of the peptides into the active site, which are not fully explained by the structure and energetics of the best docking pose alone. These docking results are consistent with the experimental finding that peptides with a C-terminal lysine have less preference for the active site of domain I, whereas peptides with a C-terminal arginine bind both domains, with a slight preference for domain I.

Another finding of the present study is that the third carboxypeptidase-like domain of human CPD is inactive as a carboxypeptidase when tested with a fluorescent substrate at a wide range of pH values, or with a wide array of peptides at pH 6.5. In no case was carboxypeptidase activity detected for the form of CPD with mutations in a key catalytic residue in domain I and II. This observation fits with previous studies on duck and *Drosophila* CPD, which tested a small number of substrates [16,22]. This observation is also consistent with the lack of conserved active site, substrate-binding, and metal-binding residues in the third domain of CPD. For example, the critical active site Glu (Glu270 using bovine CPA1 numbering) is a Tyr in the third domain of human and duck CPD. Most of the changes in active site residues found in the third domain of CPD are conserved between human and duck, suggesting that these differences are not simply random events but are important for the function of this domain. One possibility is that the third domain functions in peptide binding. While Glu270 is essential for catalytic activity, it is not required for substrate binding, and substitution of Gln for Glu permits the protein to bind peptides but not hydrolyze them. Results from our peptide docking experiments with the tetrapeptide GQKR support this hypothesis.

Three other members of the M14B subfamily of MCPs are also inactive towards standard carboxypeptidase substrates. Two of these, CPX2 [64] and AEBP1/ACLP [65] also contain Tyr in place of the critical active site Glu270, suggesting that this Tyr is important. The third inactive member of this subfamily, CPX1, does contain Glu in the comparable position but is missing other critical active site and substrate-binding residues. Other families of enzymes contain members that are considered catalytically inactive, and the functions of most of these proteins are not yet known [58–60,66]. Recently, AEBP1/ACLP was found to induce phosphorylation and nuclear translocation of Smad3, and this was dependent on TGFβ receptor binding and kinase activity [67]. Some of the active carboxypeptidases appear to have functions that are independent of their catalytic activity. For example, CPE was proposed to function as an extracellular trophic factor that protected neurons from hydrogen peroxide-, staurosporine- and glutamate-induced cell death, and this effect did not require CPE enzyme activity (although it did require amounts of CPE much higher than would be expected to be physiological) [68]. CPM was reported to be a positive allosteric modulator of the kinin B1 receptor, and this action is independent of CPM enzyme activity [69]. The emerging concept is that some carboxypeptidases have multiple functions, including both catalytic and non-catalytic roles, while the inactive members of this gene family like CPD domain III would only have non-catalytic roles.

Taken together, our results establish that both catalytically active domains of CPD play important physiological roles by cleaving a broad range of peptide and protein substrates with C-terminal basic residues or Arg and Lys in the trans-Golgi network, within the secretory and endocytic pathways and in the cell membrane. Although both active domains display a partial overlap of their substrate preferences, our results demonstrate that each active CPD domain has evolved to divergent substrate specificities conserved through evolution. Moreover, the third domain is catalytically inactive against a wide spectrum of peptides, and the biological function of this domain remains unknown.

## Supporting information

S1 Fig Relative amount of product formed by three different dansylated tripeptides incubated with various amount of purified rhCPD. Reactions containing 200 μM dansyl-Phe-Ala-Arg (filled circles), dansyl-Phe-Gly-Arg (filled squares) or dansyl-Phe-Pro-Arg (triangles/solid line) were incubated with different amounts of enzyme in a 100 mM Tris-acetate, pH 6.5, 150 mM NaCl buffer for 60 min at 37°C.
(TIF)

S2 Fig Pharmacophore models of the active sites of human CPD domains I, II and III. Acceptor and donor maps for domain I (A and D), II (B and E) and III (C and F). Hydrogen bond acceptors and donors are indicated with a red or blue contour mesh, respectively.
(TIF)

S3 Fig Alignment of human CPD (Uniprot O75976) domains I, II and III, and human CPM (Uniprot P14384). Metal binding residues, catalytic residues, and important substrate binding residues are shown in bold and boxed. Stars above the sequence indicate residues involved in zinc binding. Residues in bold and non-boxed correspond to those residues within the loop α7–β important for substrate binding. Aligned sequences from human CPD domains I, II and III correspond to residues 50–462, 496–873 and 925–1299, respectively. Sequence of human CPM corresponds to residues 18–423. The position numbers below the sequence indicate equivalent positions according to the standard numbering system for bovine CPA.
(TIF)

S4 Fig Detailed view of the pose of the arginine from GQKR peptide at the S1’ regions of CPD domains I (A) and II (B), which mainly differ in the residue that corresponds to Leu203 of bovine CPA1 and Ser198 of CPM, Ser264 in domain I and Ans678 in domain II.
(TIF)

S5 Fig Scatter plot showing the correlation between the experimental enzyme/control ratio observed in peptidomics experiments and the number of good poses found in the docking studies. (TIF)

S1 Table Non-substrates of rhCPD identified using the tryptic peptide library. (DOCX)

S2 Table Weak substrates of rhCPD identified using the tryptic peptide library. (DOCX)

S3 Table Products of rhCPD identified using the tryptic peptide library. (DOCX)

S4 Table Non-substrates identified within substrate characterization of CPD domains I and II using the tryptic peptide library. (DOCX)

S5 Table Products identified within substrate characterization of CPD domains I and II using the tryptic peptide library. (DOCX)

S6 Table Non-substrates of rhCPD identified using HEK 293T peptides. (DOCX)

S7 Table Weak substrates of rhCPD identified using HEK293T peptides. (DOCX)

S8 Table Products of rhCPD identified using HEK293T peptides. (DOCX)

S9 Table Peptide docking results. (DOCX)
