# Supplementary figures and images for: Substrate specificity of human metallocarboxypeptidase D: Comparison of the two active carboxypeptidase domains
Source: PLoS One. 2017 Nov 13;12(11):e0187778. doi: 10.1371/journal.pone.0187778 (PMC5683605; doi:10.1371/journal.pone.0187778)

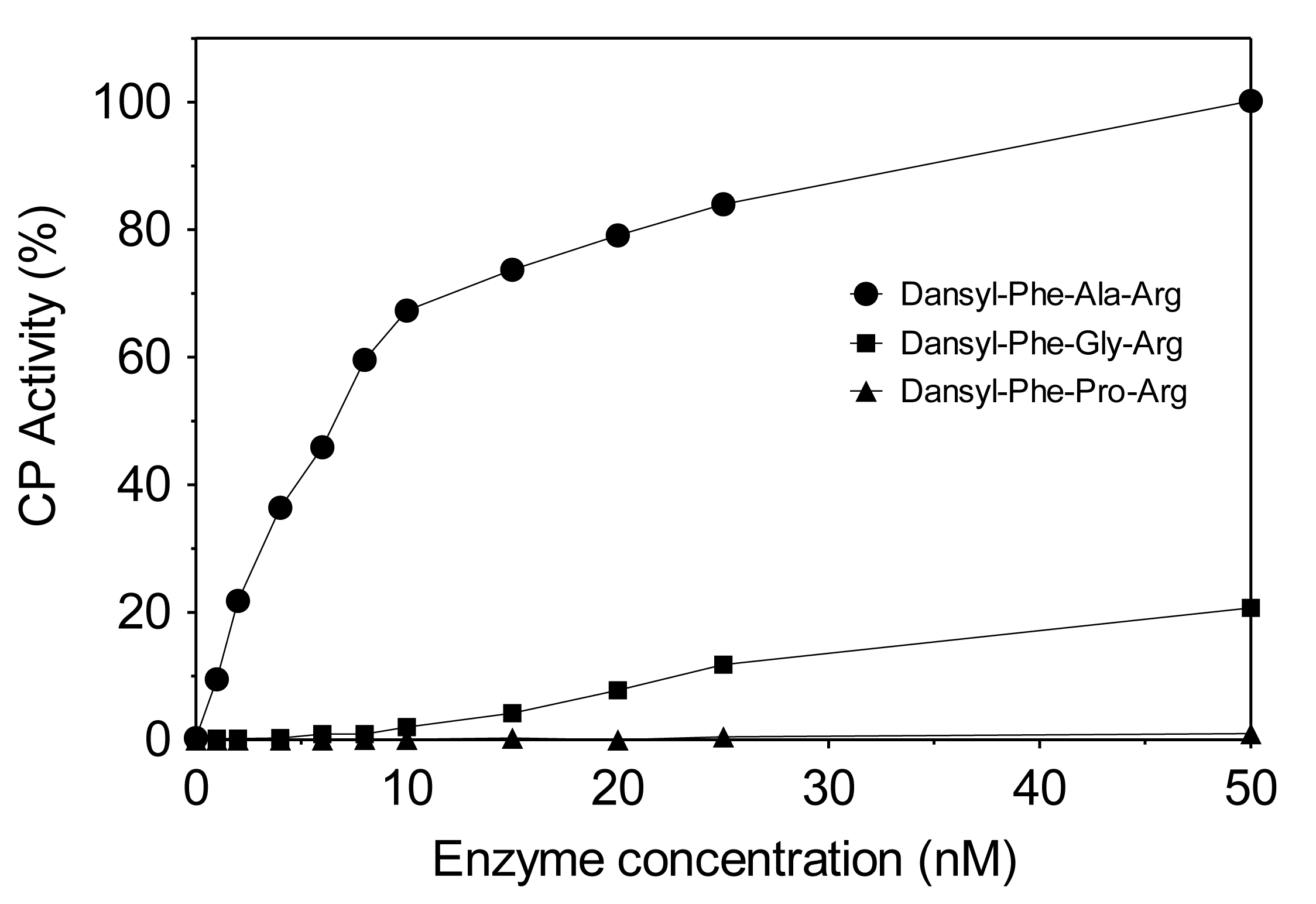

Supplement: S1 Fig — Reactions containing 200 μM dansyl-Phe-Ala-Arg (filled circles), dansyl-Phe-Gly-Arg (filled squares) or dansyl-Phe-Pro-Arg (triangles/solid line) were incubated with different amounts of enzyme in a 100 mM Tris-acetate, pH 6.5, 150 mM NaCl buffer for 60 min at 37°C. (TIF) [file pone.0187778.s001.tif]

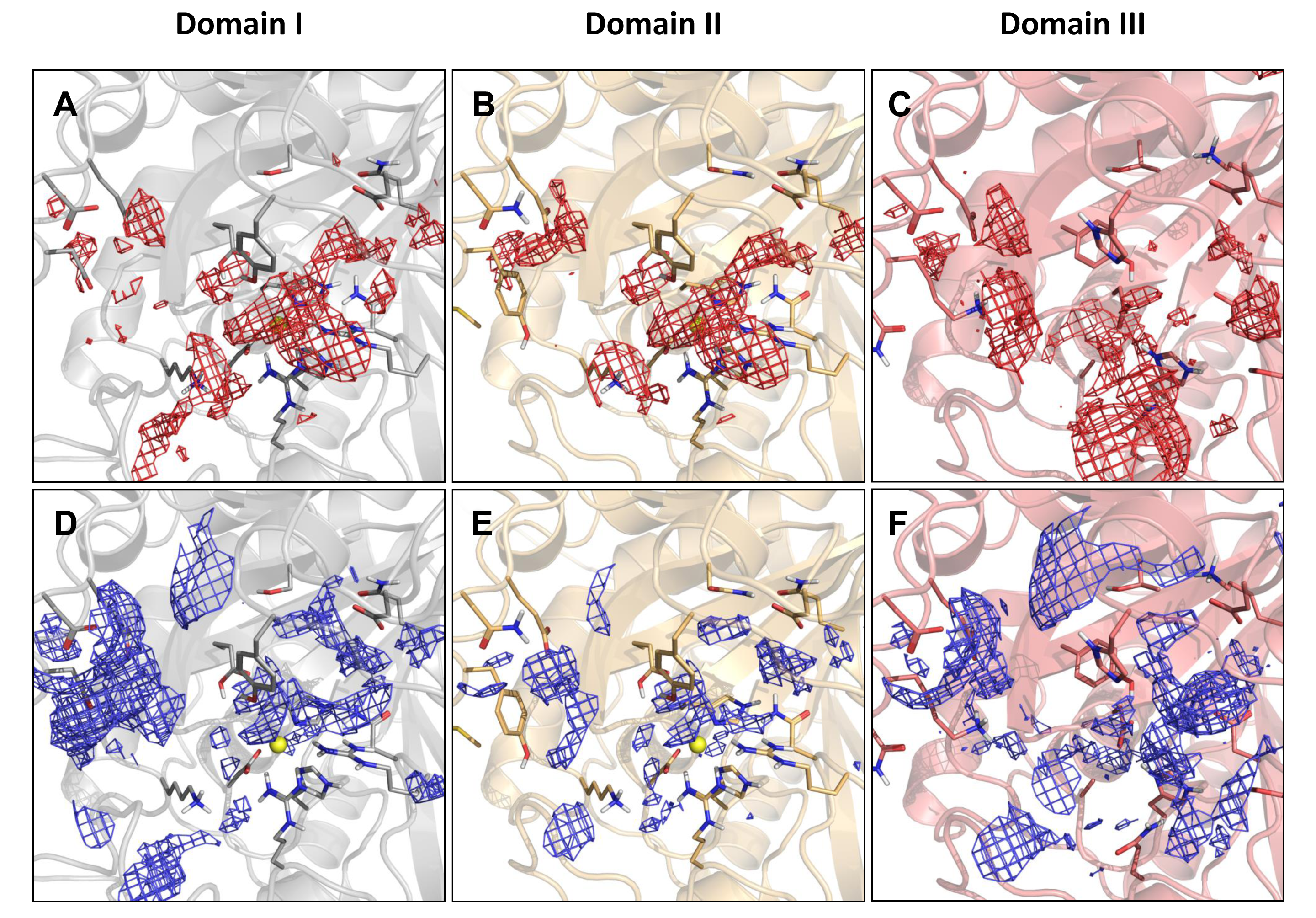

Supplement: S2 Fig — Acceptor and donor maps for domain I (A and D), II (B and E) and III (C and F). Hydrogen bond acceptors and donors are indicated with a red or blue contour mesh, respectively. (TIF) [file pone.0187778.s002.tif]

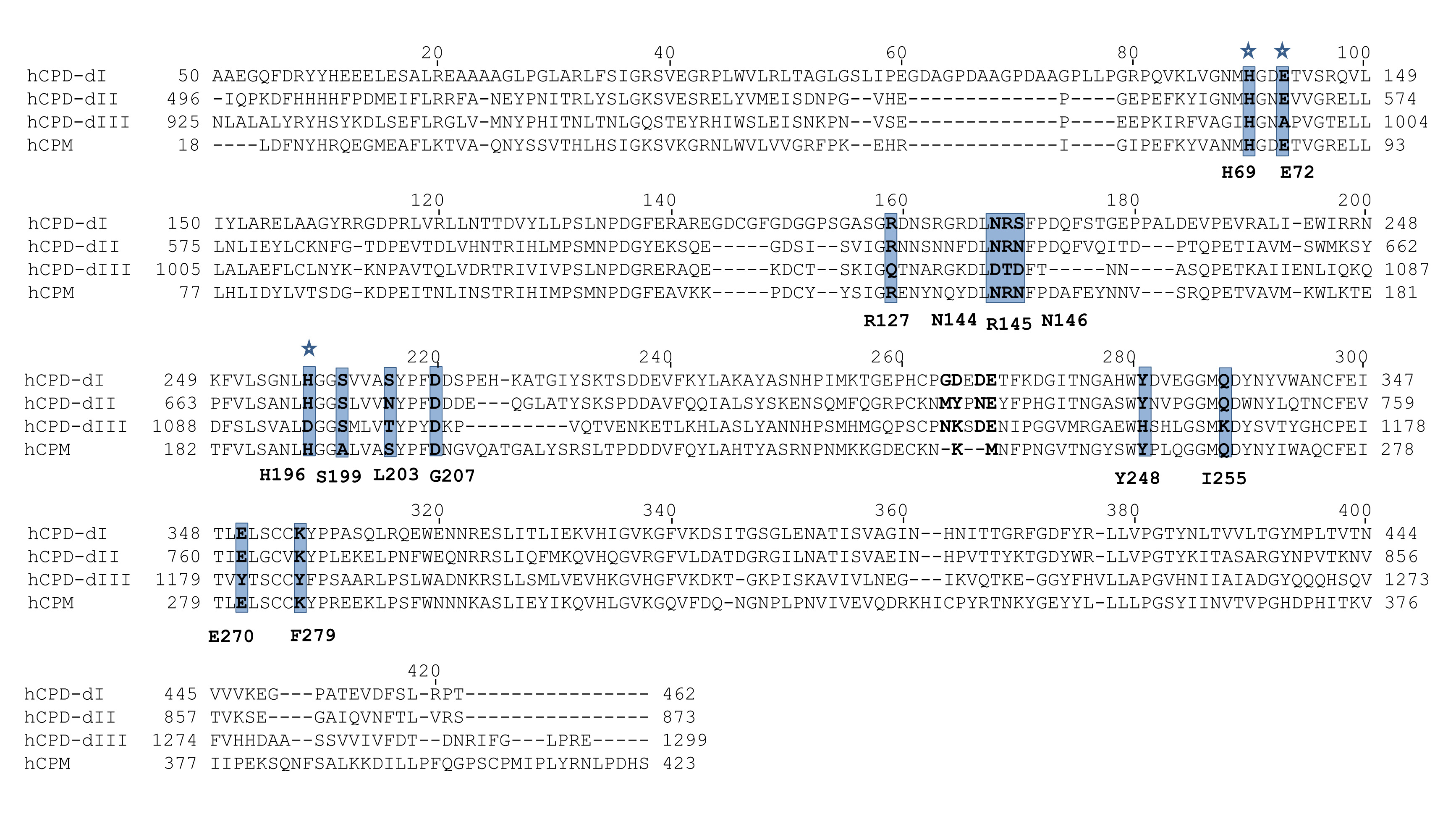

Supplement: S3 Fig — Metal binding residues, catalytic residues, and important substrate binding residues are shown in bold and boxed. Stars above the sequence indicate residues involved in zinc binding. Residues in bold and non-boxed correspond to those residues within the loop α7–β important for substrate binding. Aligned sequences from human CPD domains I, II and III correspond to residues 50–462, 496–873 and 925–1299, respectively. Sequence of human CPM corresponds to residues 18–423. The position numbers below the sequence indicate equivalent positions according to the standard numbering system for bovine CPA. (TIF) [file pone.0187778.s003.tif]

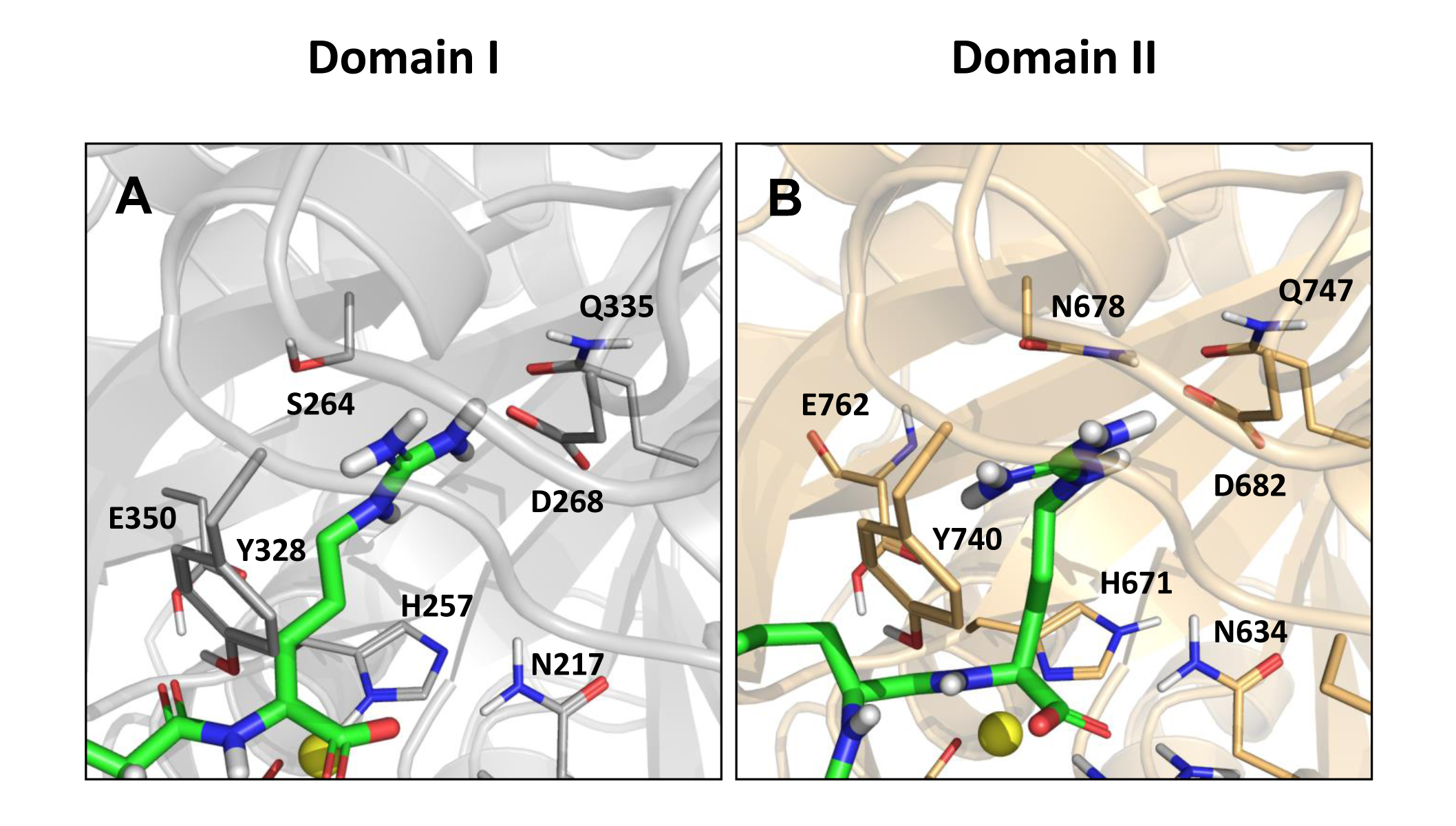

Supplement: S4 Fig — Detailed view of the pose of the arginine from GQKR peptide at the S1’ regions of CPD domains I (A) and II (B), which mainly differ in the residue that corresponds to Leu203 of bovine CPA1 and Ser198 of CPM, Ser264 in domain I and Ans678 in domain II. (TIF) [file pone.0187778.s004.tif]

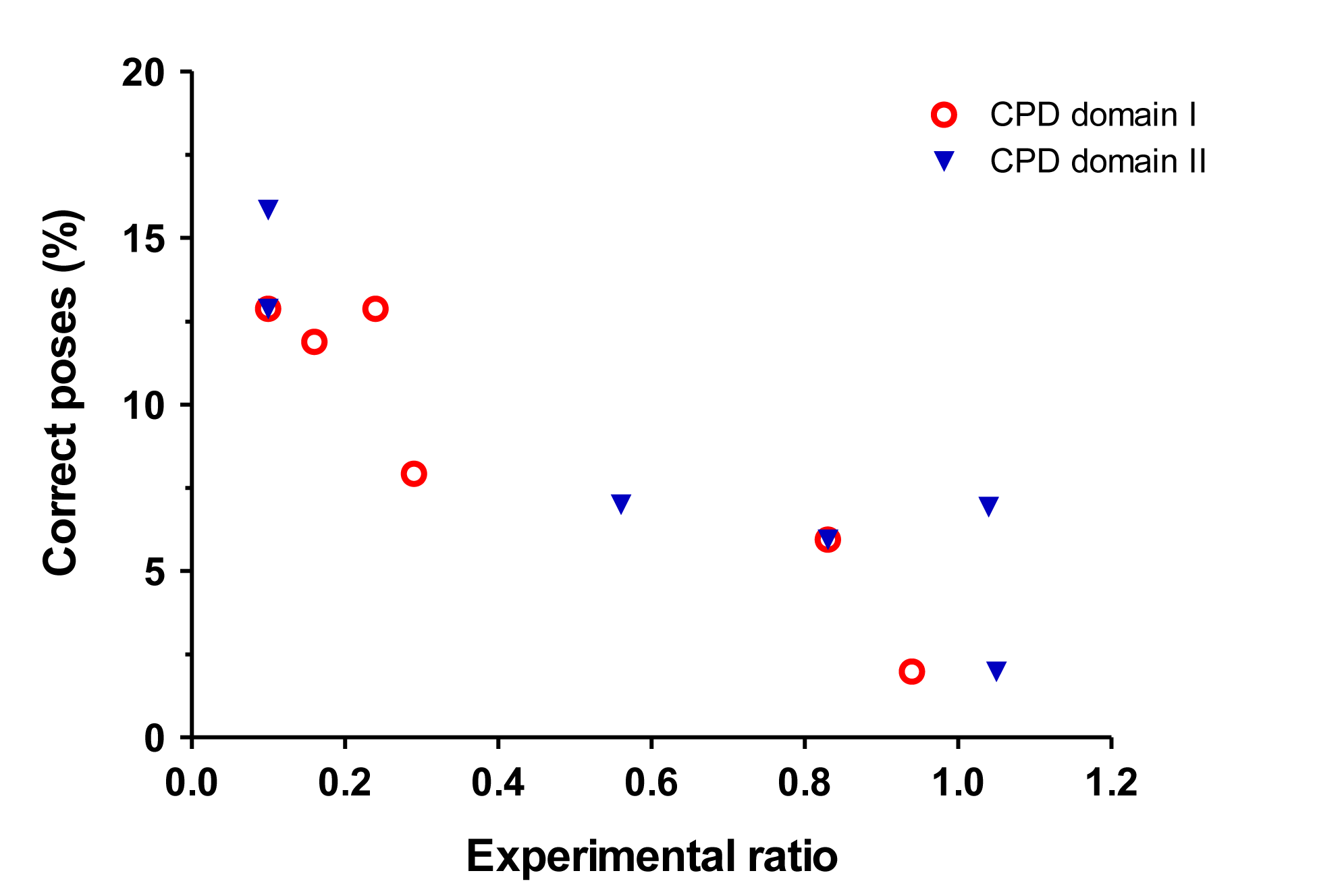

Supplement: S5 Fig — (TIF) [file pone.0187778.s005.tif]
