# Supplementary material for: Substrate specificity of human metallocarboxypeptidase D: Comparison of the two active carboxypeptidase domains
Source: PLoS One. 2017 Nov 13;12(11):e0187778. doi: 10.1371/journal.pone.0187778 (PMC5683605; doi:10.1371/journal.pone.0187778)
Supplement: S1 Table — (DOCX) [file pone.0187778.s006.docx]

| **S1** **Table. Non-substrates of rhCPD identified using the tryptic peptide library** | | | | | | | | | | |  |
| --- | --- | --- | --- | --- | --- | --- | --- | --- | --- | --- | --- |
| **Protein precursor** | **Peptide sequence** | **Z** | **T** | **Obs M** | **Theor M** | **ppm** | **Ratio rhCPD / No enzyme** | | | | |
|  |  |  |  |  |  |  | **100 nM** | **10 nM** | **1 nM** | **0.1 nM** | |
| Thyroglobulin | LPFQK | 2 | 2 | 631.37 | 631.37 | -3 | 0.82 | 0.84 | 0.98 | 1.07 | |
| Thyroglobulin | LTDEELAFPPLSPSRETFLEK | 3 | 2 | 2418.26 | 2418.23 | 12 | 0.82 | 0.85 | 1.15 | 1.09 | |
| Thyroglobulin | LQLVDAPPASLPDLQDVEEALAGK | 3 | 2 | 2488.36 | 2488.31 | 19 | 0.82 | 0.83 | 0.85 | 0.97 | |
| Thyroglobulin | GQEIPGTR | 2 | 1 | 856.45 | 856.44 | 13 | 0.83 | 0.83 | 0.9 | 0.93 | |
| Thyroglobulin | LQLVDAPPASLPDLQDVEEALAGK | 3 | 2 | 2488.36 | 2488.31 | 19 | 0.82 | 0.83 | 0.85 | 0.97 | |
| Thyroglobulin | ALADLAKP | 2 | 2 | 797.46 | 797.46 | -1 | 0.91 | 0.88 | 0.97 | 1.03 | |
| Thyroglobulin | VDLLIGSSQDDGLINR | 2 | 1 | 1713.93 | 1713.89 | 20 | 0.91 | 1.06 | 1.02 | 1.10 | |
| Thyroglobulin | AISVPEDIAR | 2 | 1 | 1069.60 | 1069.58 | 17 | 0.93 | 1.05 | 1.07 | 1.02 | |
| Thyroglobulin | SALGEPKK | 2 | 1 | 828.49 | 828.47 | 18 | 0.93 | 0.93 | 0.97 | 1.00 | |
| α-Hemoglobin | Ac-VLSPADKTNVK | 2 | 2 | 1212.68 | 1212.67 | 9 | 0.94 | 1.03 | 1.00 | 0.94 | |
| Thyroglobulin | IDVALR | 2 | 1 | 685.43 | 685.41 | 28 | 0.97 | 1.00 | 1.00 | 1.00 | |
| Thyroglobulin | LGGQEIR | 2 | 1 | 771.44 | 771.42 | 22 | 1.00 | 1.00 | 1.07 | 1.00 | |
| Bovine serum albumin | DAIPENLPPLTADFAEDK | 2 | 2 | 1954.97 | 1954.95 | 11 | 1.00 | 1.00 | 1.08 | 1.08 | |
| Bovine serum albumin | DDSPDLPK | 2 | 2 | 885.41 | 885.41 | 6 | 1.00 | 0.93 | 1.00 | 1.03 | |
| Thyroglobulin | SLLLAPEEGPVSQR | 3 | 1 | 1494.83 | 1494.80 | 23 | 1.02 | 1.15 | 1.15 | 1.24 | |
| Thyroglobulin | ALADLAKPL | 2 | 2 | 910.55 | 910.55 | 3 | 1.06 | 1.03 | 1.06 | 1.12 | |
| Thyroglobulin | KVVLQDR | 2 | 2 | 856.52 | 856.51 | 7 | 1.06 | 1.03 | 1.03 | 1.06 | |
| Thyroglobulin | LVTLAESPR | 2 | 1 | 984.58 | 984.56 | 24 | 1.07 | 1.00 | 1.07 | 1.07 | |
| Thyroglobulin | VVLQDR | 1 | 1 | 728.43 | 728.42 | 17 | 1.08 | 1.10 | 1.02 | 1.04 | |
| Thyroglobulin | RSLLLAPEEGPVSQR | 3 | 1 | 1650.94 | 1650.91 | 17 | 1.08 | 1.10 | 1.14 | 1.12 | |
| Thyroglobulin | QAGVQAEPSPK | 3 | 2 | 1110.57 | 1110.57 | 3 | 1.09 | 0.99 | 0.98 | 1.11 | |
| Bovine serum albumin | LVVSTQTALA | 2 | 1 | 1001.60 | 1001.58 | 16 | 1.10 | 1.10 | 1.10 | 1.00 | |
| Thyroglobulin | ASGLGAAAGQR | 2 | 1 | 957.52 | 957.50 | 18 | 1.10 | 1.07 | 1.10 | 1.07 | |
| Thyroglobulin | FLQGDR | 1 | 1 | 734.39 | 734.37 | 22 | 1.11 | 1.01 | 1.07 | 1.07 | |
| Thyroglobulin | LNSNPASEAPK | 2 | 2 | 1126.57 | 1126.56 | 5 | 1.12 | 0.97 | 1.06 | 1.03 | |
| Thyroglobulin | LQQNLFGGR | 2 | 1 | 1031.57 | 1031.55 | 19 | 1.12 | 1.12 | 1.04 | 1.12 | |
| Thyroglobulin | VTLAADR | 1 | 1 | 744.43 | 744.41 | 30 | 1.14 | 1.16 | 1.16 | 1.09 | |
| Trypsin^1^ | VATVSLPR | 2 | 1 | 841.52 | 841.50 | 19 | 1.14 | 1.25 | 1.18 | 1.07 | |
| Thyroglobulin | ETFLEK | 2 | 2 | 765.39 | 765.39 | 3 | 1.14 | 1.07 | 1.18 | 1.14 | |
| Thyroglobulin | FAATSFR | 2 | 1 | 798.42 | 798.40 | 24 | 1.15 | 1.15 | 1.11 | 1.07 | |
| ^1^Fragment originated from trypsin autolysis. See Table 2 for the abbreviation definitions. | | | | | | | | | | | |
