# Supplementary material for: Substrate specificity of human metallocarboxypeptidase D: Comparison of the two active carboxypeptidase domains
Source: PLoS One. 2017 Nov 13;12(11):e0187778. doi: 10.1371/journal.pone.0187778 (PMC5683605; doi:10.1371/journal.pone.0187778)
Supplement: S2 Table — (DOCX) [file pone.0187778.s007.docx]

| **S2 Table**. **Weak substrates of rhCPD identified using the tryptic peptide library** | | | | | | | | | | |  |
| --- | --- | --- | --- | --- | --- | --- | --- | --- | --- | --- | --- |
| **Protein precursor** | **Peptide sequence** | **Z** | **T** | **Obs M** | **Theor M** | **ppm** | **Ratio rhCPD / No enzyme** | | | | |
|  |  |  |  |  |  |  | **100 nM** | **10 nM** | **1 nM** | **0.1 nM** | |
| Thyroglobulin | FEKLPESK | 3 | 3 | 976.53 | 976.52 | 8 | 0.42 | 1.00 | 0.97 | 1.15 | |
| Thyroglobulin | KGQEFTITGQK | 3 | 3 | 1235.66 | 1235.65 | 7 | 0.53 | 0.67 | 0.86 | 1.11 | |
| Thyroglobulin | ALEQATR | 2 | 1 | 787.43 | 787.42 | 19 | 0.55 | 0.65 | 0.81 | 0.90 | |
| Thyroglobulin | FVAPESLK | 2 | 2 | 889.50 | 889.49 | 10 | 0.61 | 0.79 | 1.00 | 1.03 | |
| Thyroglobulin | ILNDAQTK | 2 | 2 | 901.49 | 901.49 | 5 | 0.67 | 0.90 | 1.10 | 1.10 | |
| Bovine serum albumin | AEFVEVTK | 1 | 2 | 921.50 | 921.48 | 18 | 0.67 | 0.91 | 0.97 | 0.99 | |
| Bovine serum albumin | KVPQVSTPTLVEVSR | 3 | 2 | 1638.95 | 1638.93 | 14 | 0.68 | 0.92 | 1.00 | 1.08 | |
| Bovine serum albumin | KQTALVELLK | 3 | 3 | 1141.70 | 1141.71 | -6 | 0.73 | 1.00 | 1.14 | 1.00 | |
| α-Hemoglobin | VLSPADKTNVK | 3 | 3 | 1170.67 | 1170.66 | 5 | 0.74 | 0.86 | 1.00 | 1.08 | |
| Thyroglobulin | AVKQFEESQGR | 2 | 2 | 1277.66 | 1277.64 | 17 | 0.76 | 0.78 | 0.86 | 1.12 | |
| Bovine serum albumin | VPQVSTPTLVEVSR | 3 | 1 | 1510.87 | 1510.84 | 22 | 0.77 | 1.03 | 1.08 | 1.08 | |
| Thyroglobulin | ELSVLLPNR | 2 | 1 | 1039.62 | 1039.60 | 2 | 0.79 | 0.97 | 1.06 | 1.12 | |
| β-Hemoglobin | VNVDEVGGEALGR | 2 | 1 | 1313.69 | 1313.66 | 19 | 0.79 | 0.91 | 1.03 | 1.06 | |
| Weak substrates, peptides affected with a decrease ≥20% and <60% by the highest concentration of enzyme. See Table 2 for abbreviation definitions. | | | | | | | | | | | |
