# Supplementary material for: Substrate specificity of human metallocarboxypeptidase D: Comparison of the two active carboxypeptidase domains
Source: PLoS One. 2017 Nov 13;12(11):e0187778. doi: 10.1371/journal.pone.0187778 (PMC5683605; doi:10.1371/journal.pone.0187778)
Supplement: S3 Table — (DOCX) [file pone.0187778.s008.docx]

| **S3 Table. Products of rhCPD identified using the tryptic peptide library** | | | | | | | | | | | |  |
| --- | --- | --- | --- | --- | --- | --- | --- | --- | --- | --- | --- | --- |
| **Protein precursor** | **Peptide sequence** | **Cleaved aa** | **Z** | **T** | **Obs M** | **Theor M** | **ppm** | **Ratio rhCPD / No enzyme** | | | | |
|  |  |  |  |  |  |  |  | **100 nM** | **10 nM** | **1 nM** | **0.1 nM** | |
| Thyroglobulin | LF | R | 1 | 1 | 278.16 | 278.15 | 30 | 3.63 | 1.75 | 0.88 | 0.88 | |
| Bovine serum albumin | AEFVEVT | K | 2 | 1 | 793.40 | 793.39 | 13 | >5.00 | ND | ND | ND | |
| Bovine serum albumin | LVNELTEFA | K | 1 | 1 | 1034.54 | 1034.53 | 12 | >5.00 | ND | ND | ND | |
| Products, peptides with an increase >120% with one or more concentrations of enzyme; Cleaved aa, the amino acid cleaved by rhCPD to generate the observed peptide; ND, not detectable. See Table 2 for the rest of abbreviation definitions. | | | | | | | | | | | | |
