# Supplementary material for: Substrate specificity of human metallocarboxypeptidase D: Comparison of the two active carboxypeptidase domains
Source: PLoS One. 2017 Nov 13;12(11):e0187778. doi: 10.1371/journal.pone.0187778 (PMC5683605; doi:10.1371/journal.pone.0187778)
Supplement: S4 Table — (DOCX) [file pone.0187778.s009.docx]

| **S4 Table. Non-substrates identified within substrate characterization of CPD domains I and II using the tryptic peptide library** | | | | | | | | | |  |
| --- | --- | --- | --- | --- | --- | --- | --- | --- | --- | --- |
| **Protein precursor** | **Peptide sequence** | **Z** | **T** | **Obs M** | **Theor M** | **ppm** | **Ratio enzyme / Control** | | | |
|  |  |  |  |  |  |  | **rhCPD** | **Domain I active** | **Domain II active** | |
| Thyroglobulin | LILPR | 1 | 1 | 610.44 | 610.42 | 37 | 0.88 | 0.85 | 0.91 | |
| Thyroglobulin | ALADLAKPL | 2 | 2 | 910.55 | 910.55 | -3 | 0.88 | 0.88 | 0.92 | |
| Thyroglobulin | ALADLAKPLS | 2 | 2 | 997.59 | 997.58 | 7 | 0.88 | 0.90 | 0.82 | |
| Thyroglobulin | RLVTLAESPR | 3 | 1 | 1140.69 | 1140.66 | 22 | 0.89 | 0.98 | 1.00 | |
| Thyroglobulin | VTLAADR | 2 | 1 | 744.42 | 744.41 | 8 | 0.90 | 0.95 | 1.05 | |
| Bovine serum albumin | LKPDPNTL | 2 | 2 | 896.49 | 896.50 | -3 | 0.92 | 1.20 | 1.09 | |
| Thyroglobulin | RVTLAADR | 3 | 1 | 900.51 | 900.51 | -5 | 0.92 | 1.01 | 0.84 | |
| Thyroglobulin | LVTLAESPR | 2 | 1 | 984.59 | 984.56 | 35 | 0.92 | 0.96 | 0.92 | |
| Thyroglobulin | ALADLAKP | 2 | 2 | 797.44 | 797.46 | -32 | 0.92 | 0.92 | 0.87 | |
| Thyroglobulin | ILNDAQTK | 2 | 2 | 901.49 | 901.49 | 1 | 0.94 | 1.20 | 0.65 | |
| Thyroglobulin | FVAPESLK | 2 | 2 | 889.48 | 889.49 | -11 | 0.94 | 1.18 | 0.82 | |
| Thyroglobulin | FARFTASCPPSIK | 2 | 2 | 1423.78 | 1423.73 | 38 | 0.95 | 0.99 | 0.92 | |
| α-Hemoglobin | VLSPADKTNVK | 2 | 3 | 1170.68 | 1170.66 | 13 | 0.96 | 1.13 | 0.78 | |
| Thyroglobulin | IDVALR | 2 | 1 | 685.40 | 685.41 | -9 | 0.98 | 0.84 | 0.90 | |
| Bovine serum albumin | DDSPDLPK | 2 | 2 | 885.40 | 885.41 | -15 | 1.03 | 1.18 | 1.32 | |
| Thyroglobulin | VLQFIR | 2 | 1 | 774.48 | 774.48 | 12 | 1.04 | 0.98 | 0.94 | |
| α-Hemoglobin | Ac-VLSPADKTNVK | 2 | 2 | 1212.65 | 1212.67 | -14 | 1.05 | 1.19 | 0.83 | |
| Thyroglobulin | ETFLEK | 2 | 2 | 765.37 | 765.39 | -25 | 1.05 | 1.26 | 0.95 | |
| Thyroglobulin | LGGQEIR | 2 | 1 | 771.43 | 771.42 | 13 | 1.06 | 1.06 | 1.11 | |
| Thyroglobulin | SALGEPKK | 2 | 1 | 828.48 | 828.47 | 14 | 1.08 | 1.04 | 0.94 | |
| Thyroglobulin | GQEFTITGQK | 2 | 2 | 1107.59 | 1107.56 | 24 | 1.08 | 1.18 | 0.98 | |
| Trypsin^1^ | VATVSLPR | 2 | 1 | 841.52 | 841.50 | 21 | 1.09 | 0.97 | 0.97 | |
| Thyroglobulin | LQQNLFGGR | 2 | 1 | 1031.60 | 1031.55 | 46 | 1.09 | 0.65 | 1.09 | |
| Thyroglobulin | ILQR | 1 | 1 | 528.34 | 528.34 | -4 | 1.10 | 0.74 | 1.18 | |
| Thyroglobulin | FLQGDR | 2 | 1 | 734.38 | 734.37 | 7 | 1.10 | 1.03 | 1.05 | |
| Thyroglobulin | LNSNPASEAPK | 2 | 2 | 1126.58 | 1126.56 | 20 | 1.11 | 0.94 | 1.11 | |
| Thyroglobulin | QAGVQAEPSPK | 2 | 2 | 1110.60 | 1110.57 | 25 | 1.11 | 1.05 | 1.05 | |
| Thyroglobulin | GLFPSR | 2 | 1 | 675.36 | 675.37 | -12 | 1.12 | 1.02 | 1.15 | |
| Thyroglobulin | LTGISIR | 2 | 1 | 758.47 | 758.47 | 8 | 1.13 | 1.02 | 1.06 | |
| ^1^Fragment originated from trypsin autolysis. See Table 2 for the abbreviation definitions. Control: Reaction in absence of enzyme. | | | | | | | | | | |
