# Supplementary material for: Substrate specificity of human metallocarboxypeptidase D: Comparison of the two active carboxypeptidase domains
Source: PLoS One. 2017 Nov 13;12(11):e0187778. doi: 10.1371/journal.pone.0187778 (PMC5683605; doi:10.1371/journal.pone.0187778)
Supplement: S5 Table — (DOCX) [file pone.0187778.s010.docx]

| **S5 Table.** **Products identified within substrate characterization of CPD domains I and II using the tryptic peptide library** | | | | | | | | | | | |  |
| --- | --- | --- | --- | --- | --- | --- | --- | --- | --- | --- | --- | --- |
| **Protein precursor** | **Peptide sequence** |  | **Z** | **T** | **Obs M** | **Theor M** | **Ppm** | **Ratio enzyme^1^ / Control^2^** | | | **Ratio dI active / d II active** | |
|  |  |  |  |  |  |  |  | **rhCPD** | **Domain I active** | **Domain II active** |  |  |
| Thyroglobulin | GLFPS | R | 1 | 1 | 519.26 | 519.26 | 12 | >5.00 | >5.00 | >5.00 | 1.08 | |
| Thyroglobulin | AFLGTV | R | 1 | 1 | 606.34 | 606.33 | 29 | >5.00 | >5.00 | >5.00 | 0.89 | |
| Thyroglobulin | FAATSF | R | 1 | 1 | 642.31 | 642.29 | 36 | >5.00 | >5.00 | >5.00 | 0.88 | |
| α-Hemoglobin | VLSPADKTNV | K | 2 | 2 | 1042.59 | 1042.57 | 23 | >5.00 | 0.59 | >5.00 | <0.10 | |
| α-Hemoglobin | LRVDPVNF | K | 2 | 1 | 958.56 | 958.52 | 40 | >5.00 | 2.35 | >5.00 | <0.10 | |
| Thyroglobulin | FEKLPES | K | 2 | 2 | 848.42 | 848.43 | -5 | >5.00 | 0.59 | >5.00 | <0.10 | |
| Products, peptides with an increase >120% with one or more concentrations of enzyme. Cleaved aa, the amino acid cleaved by rhCPD to generate the observed peptide. See Table 2 and 3 for the rest of abbreviation definitions. | | | | | | | | | | | | |
