# Supplementary material for: Substrate specificity of human metallocarboxypeptidase D: Comparison of the two active carboxypeptidase domains
Source: PLoS One. 2017 Nov 13;12(11):e0187778. doi: 10.1371/journal.pone.0187778 (PMC5683605; doi:10.1371/journal.pone.0187778)
Supplement: S6 Table — (DOCX) [file pone.0187778.s011.docx]

| **S6 Table. Non-substrates of rhCPD identified using HEK 293T peptides** | | | | | | | | | | | | |  |
| --- | --- | --- | --- | --- | --- | --- | --- | --- | --- | --- | --- | --- | --- |
| **Precursor** | | **Sequence** | **Z** | **T** | **Obs M** | **Theor M** | **ppm** | **Ratio rhCPD / No enzyme** | | | | | |
|  |  |  |  |  |  |  |  | **100 nM** | **10 nM** | **1 nM** | | **0.1 nM** | |
| 40S Ribosomal protein S28 | Ac-MDTSRVQPIKLA | | 2 | 1 | 1399.79 | 1399.749 | 30 | 0.82 | 1.08 | 1.00 | | 0.97 | |
| 40S Ribosomal protein S21 | KADGIVSKNF | | 3 | 3 | 1077.59 | 1077.582 | 4 | 0.84 | 0.95 | 0.95 | | 0.70 | |
| 60S acidic ribosomal protein P2 polymorphism | DGKNIEDVIAQGIGKL | | 3 | 3 | 1668.92 | 1668.905 | 10 | 0.84 | 0.89 | 0.92 | | 0.74 | |
| Heat shock 10kDa protein | AAETVTKGGIMLPEKSQGKVLQA | | 4 | 4 | 2355.32 | 2355.283 | 15 | 0.86 | 1.10 | 1.04 | | 1.08 | |
| Heat shock 10kDa protein | GSGSKGKGGEIQPVSV | | 3 | 3 | 1485.79 | 1485.779 | 7 | 0.91 | 0.98 | 0.94 | | 0.91 | |
| Superoxide dismutase 1 | KGDGPVQGIINF | | 2 | 2 | 1243.67 | 1243.656 | 14 | 0.92 | 0.96 | 0.94 | | 0.89 | |
| Prefoldin subunit 1 | Ac-AAPVDLELKKAFTEL | | 2 | 2 | 1685.96 | 1685.924 | 22 | 0.92 | 0.92 | 0.95 | | 0.99 | |
| Heterogeneous nuclear ribonucleoprotein D-like | KDAASVDKVLEL | | 3 | 3 | 1286.72 | 1286.708 | 7 | 0.93 | 0.89 | 0.97 | | 0.95 | |
| Triosephosphate isomerase 1 | SLGELIGTLNA | | 2 | 1 | 1086.62 | 1086.592 | 22 | 0.94 | 0.90 | 0.94 | | 0.81 | |
| Heat shock 10kDa protein 1 | LPLFDRVLVE | | 2 | 1 | 1199.72 | 1199.691 | 25 | 0.94 | 1.04 | 1.00 | | 1.13 | |
| Triosephosphate isomerase 1 | LDPKIAVA | | 2 | 2 | 825.50 | 825.496 | 3 | 0.94 | 1.06 | 0.94 | | 0.94 | |
| Ubiquitin-60S ribosomal protein L40 | IIEPSLRQL | | 2 | 1 | 1067.66 | 1067.634 | 21 | 0.94 | 0.97 | 0.94 | | 0.89 | |
| 60S Ribosomal protein L31 | KNLQTVNVDEN | | 2 | 2 | 1272.64 | 1272.631 | 8 | 0.94 | 1.00 | 0.94 | | 0.94 | |
| Elongation factor 1 beta | GFGDLKSPAGLQVL | | 2 | 2 | 1400.79 | 1400.770 | 12 | 0.95 | 1.05 | 0.89 | | 1.03 | |
| Vimentin | LIKTVETRDGQVINETSQ | | 3 | 2 | 2030.11 | 2030.064 | 23 | 0.95 | 1.10 | 0.95 | | 0.90 | |
| FK506 Binding Protein | VFDVELL | | 2 | 1 | 833.47 | 833.453 | 21 | 0.95 | 0.95 | 0.95 | | 0.92 | |
| Nucleophosmin | ASIEKGGSLPKVEA | | 2 | 3 | 1384.76 | 1384.756 | 0 | 0.97 | 0.97 | 0.91 | | 0.86 | |
| Cytochrome c oxidase subunit 5a | GISTPEELGLDKV | | 2 | 2 | 1356.73 | 1356.714 | 14 | 0.97 | 1.06 | 0.95 | | 0.95 | |
| Peptidylprolyl isomerase A | ADKVPKTAENFRAL | | 3 | 3 | 1558.86 | 1558.847 | 8 | 0.97 | 1.09 | 0.99 | | 0.99 | |
| Complement component 1 Q subcomponent-binding protein, mitocondrial | DRGVDNTFADELVELSTA | | 2 | 1 | 1950.96 | 1950.917 | 20 | 0.98 | 1.07 | 1.07 | | 0.86 | |
| Heterogeneous nuclear ribonucleoprotein A/B isoform 1 | FGEFGEIEAIEL | | 2 | 1 | 1352.69 | 1352.650 | 29 | 1.00 | 1.15 | 1.13 | | 0.88 | |
| Nucleophosmin | GGFEITPPVVL | | 2 | 1 | 1127.65 | 1127.623 | 23 | 1.00 | 1.06 | 1.03 | | 0.94 | |
| Peptidylprolyl isomerase A | ELFADKVPKTA | | 3 | 3 | 1217.67 | 1217.666 | 4 | 1.00 | 1.10 | 1.05 | | 0.95 | |
| Heat shock 10kDa protein | TVVAVGSGSKGKGGEIQPV | | 3 | 3 | 1768.99 | 1768.968 | 13 | 1.00 | 1.09 | 0.85 | | 0.96 | |
| FK506 Binding Protein | VFDVELLKLE | | 2 | 2 | 1203.70 | 1203.675 | 17 | 1.00 | 0.97 | 0.97 | | 0.96 | |
| Peptidylprolyl isomerase A | ADKVPKTAENF | | 3 | 3 | 1218.62 | 1218.624 | -3 | 1.01 | 0.99 | 0.99 | | 0.89 | |
| Nucleophosmin | GGSLPKVEA | | 2 | 2 | 856.47 | 856.465 | 9 | 1.03 | 1.11 | 0.97 | | 0.87 | |
| 40S Ribosomal protein S21 | AKADGIVSKNF | | 2 | 3 | 1148.62 | 1148.619 | -1 | 1.04 | 1.05 | 0.95 | | 0.93 | |
| 40S Ribosomal protein S21 | ADGIVSKNF | | 2 | 2 | 949.49 | 949.487 | 8 | 1.04 | 1.04 | 1.00 | | 0.96 | |
| Elongation factor 1 beta | GFGDLKSPAGL | | 2 | 2 | 1060.57 | 1060.555 | 11 | 1.04 | 1.10 | | 1.00 | 1.12 | |
| FK506 Binding Protein | GVQVETISPGDGRTFPKRGQ | | 4 | 2 | 2128.16 | 2128.102 | 25 | 1.05 | 1.07 | | 1.03 | 1.02 | |
| Heterogeneous nuclear ribonucleoprotein D0 | FGGFGEVESIEL | | 2 | 1 | 1282.64 | 1282.608 | 27 | 1.06 | 1.03 | | 0.89 | 0.89 | |
| RNA binding motif protein 3 | Ac-SSEEGKLFVGGLNF | | 2 | 1 | 1524.78 | 1524.746 | 25 | 1.06 | 1.06 | | 1.06 | 0.94 | |
| Peptidylprolyl isomerase A | ELFADKVPKTAENFRAL | | 4 | 3 | 1948.05 | 1948.042 | 5 | 1.06 | 1.09 | | 1.06 | 1.12 | |
| FK506 Binding Protein | VELLKLE | | 2 | 2 | 842.52 | 842.511 | 6 | 1.06 | 1.04 | | 1.00 | 1.00 | |
| Eukaryotic translation initiation factor 4H | ATPLNQVANPNSAIFGGARPREEVVQKEQE | | 4 | 2 | 3248.73 | 3248.654 | 24 | 1.07 | 1.13 | | 1.00 | 0.93 | |
| Protein DJ-1 (Parkinson disease protein 7) | APLVLKD | | 2 | 2 | 754.46 | 754.459 | -3 | 1.07 | 1.00 | | 0.93 | 1.07 | |
| 60S acidic ribosomal protein P2 | VGIEADDDRLNKV | | 3 | 2 | 1442.76 | 1442.736 | 13 | 1.07 | 1.10 | | 1.04 | 1.10 | |
| Elongation factor 1 beta | GFGDLKSPAGLQV | | 3 | 2 | 1287.70 | 1287.682 | 14 | 1.07 | 1.12 | | 1.06 | 1.13 | |
| Peptidylprolyl isomerase A | VNPTVFFDI | | 2 | 1 | 1050.57 | 1050.539 | 26 | 1.08 | 1.06 | | 1.00 | 1.00 | |
| 40S Ribosomal protein S29 | AKDIGFIKLD | | 3 | 3 | 1118.64 | 1118.634 | 3 | 1.08 | 1.06 | | 1.00 | 1.00 | |
| Protein SET (Phosphatase 2A inhibitor I2PP2A) - isoform 2 | Ac-SAPAAKVSKKEL | | 2 | 3 | 1269.73 | 1269.729 | 3 | 1.09 | 1.13 | | 1.06 | 1.15 | |
| Heat shock 10kDa protein 1 | AVGSGSKGKGGEIQPVSV | | 3 | 3 | 1655.89 | 1655.884 | 2 | 1.09 | 1.03 | | 0.99 | 0.92 | |
| Heterogeneous nuclear ribonucleoprotein D-like | ASVDKVLEL | | 2 | 2 | 972.56 | 972.549 | 9 | 1.10 | 1.14 | | 1.00 | 1.14 | |
| Heat shock 10kDa protein | VAVGSGSKGKGGEIQPVSV | | 3 | 3 | 1754.97 | 1754.953 | 11 | 1.11 | 1.06 | | 0.83 | 0.98 | |
| Nucleophosmin | EKGGSLPKVEA | | 3 | 3 | 1113.60 | 1113.603 | -5 | 1.12 | 1.12 | | 1.12 | 0.86 | |
| Heat shock 10kDa protein 1 | TVVAVGSGSKGKGGEIQPVSV | | 4 | 3 | 1955.07 | 1955.069 | 2 | 1.12 | 1.08 | | 0.98 | 0.88 | |
| Complement component 1 Q subcomponent-binding protein, mitocondrial | ADRGVDNTFADELVEL | | 2 | 1 | 1762.88 | 1762.837 | 24 | 1.14 | 1.08 | | 0.97 | 1.00 | |
| Heat shock 10kDa protein 1 | VGSGSKGKGGEIQPVSV | | 3 | 3 | 1584.85 | 1584.847 | 2 | 1.14 | 1.09 | | 1.00 | 1.09 | |
| Protein SET (Phosphatase 2A inhibitor I2PP2A) - isoform 1 | SELIAKI | | 2 | 2 | 772.47 | 772.469 | 3 | 1.14 | 1.00 | | 1.00 | 0.86 | |
| 40S ribosomal protein S12 | Ac-AEEGIAAGGVMDVNTALQEVLKT | | 3 | 1 | 2357.26 | 2357.178 | 35 | 1.15 | 1.13 | | 1.15 | 1.06 | |
| 40S Ribosomal protein S28 | Ac-MoxDTSRVQPIKL | | 2 | 1 | 1344.74 | 1344.712 | 19 | 1.16 | 1.04 | | 0.98 | 0.98 | |
| See Table 2 for the abbreviation definitions. | | | | | | | | | | | | | |
