# Supplementary material for: Substrate specificity of human metallocarboxypeptidase D: Comparison of the two active carboxypeptidase domains
Source: PLoS One. 2017 Nov 13;12(11):e0187778. doi: 10.1371/journal.pone.0187778 (PMC5683605; doi:10.1371/journal.pone.0187778)
Supplement: S7 Table — (DOCX) [file pone.0187778.s012.docx]

| **S7 Table. Weak substrates of rhCPD identified using HEK293T peptides** | | | | | | | | | | |  |
| --- | --- | --- | --- | --- | --- | --- | --- | --- | --- | --- | --- |
| **Precursor** | **Sequence** | **Z** | **T** | **Obs M** | **Theor M** | **ppm** | **Ratio rhCPD / No enzyme** | | | | |
|  |  |  |  |  |  |  | **100 nM** | **10 nM** | **1 nM** | **0.1 nM** | |
| Hematological and neurol. exp. 1 prot. | Ac-TTTTTFKGVDPNSRNSSR | 3 | 1 | 2010.02 | 2009.977 | 20 | 0.40 | 1.13 | 1.07 | 0.97 | |
| Cathepsin D | GPIPEVLK | 2 | 2 | 851.51 | 851.512 | 1 | 0.58 | 0.99 | 0.95 | 0.94 | |
| CD99 antigen | AEPAVQRTLLEK | 3 | 2 | 1353.77 | 1353.762 | 10 | 0.61 | 0.95 | 0.99 | 0.97 | |
| Ubiquitin-60S ribosomal protein L40 | IIEPSLR | 2 | 1 | 826.51 | 826.491 | 18 | 0.73 | 1.03 | 1.00 | 1.08 | |
| Weak substrates, peptides affected with a decrease ≥20% and <60% by the highest concentration of enzyme. See Table 2 for abbreviation definitions. | | | | | | | | | | | |
