# Supplementary material for: Substrate specificity of human metallocarboxypeptidase D: Comparison of the two active carboxypeptidase domains
Source: PLoS One. 2017 Nov 13;12(11):e0187778. doi: 10.1371/journal.pone.0187778 (PMC5683605; doi:10.1371/journal.pone.0187778)
Supplement: S8 Table — (DOCX) [file pone.0187778.s013.docx]

| **S8** **Table.** **Products of rhCPD identified using HEK293T peptides** | | | | | | | | | | | |  |
| --- | --- | --- | --- | --- | --- | --- | --- | --- | --- | --- | --- | --- |
| **Precursor** | **Sequence** | **Cleaved aa** | **Z** | **T** | **Obs M** | **Theor M** | **ppm** | **Ratio rhCPD / No enzyme** | | | | |
|  |  |  |  |  |  |  |  | **100 nM** | **10 nM** | **1 nM** | **0.1 nM** | |
| 40S Ribosomal protein S28 | Ac-MoxDTSRVQPIKLA | R | 3 | 1 | 1415.78 | 1415.749 | 25 | 1.56 | 1.12 | 1.21 | 1.02 | |
| Nucleophosmin | EKTPKTPKGPSSVEDIKA | K | 5 | 5 | 1911.03 | 1911.03 | -2 | 1.86 | 1.39 | 1.15 | 1.11 | |
| Products, peptides with an increase >120% with one or more concentrations of enzyme. Cleaved aa, the amino acid cleaved by rhCPD to generate the observed peptide. See Table 2 for the rest of abbreviation definitions. | | | | | | | | | | | | |
