# Supplementary material for: Substrate specificity of human metallocarboxypeptidase D: Comparison of the two active carboxypeptidase domains
Source: PLoS One. 2017 Nov 13;12(11):e0187778. doi: 10.1371/journal.pone.0187778 (PMC5683605; doi:10.1371/journal.pone.0187778)
Supplement: S9 Table — (DOCX) [file pone.0187778.s014.docx]

| **S9 Table. Peptide docking results** | | | | | | | | | |
| --- | --- | --- | --- | --- | --- | --- | --- | --- | --- |
| **Peptide sequence** | **CPD domain I** | |  | **CPD domain II** | | | |  |  |
|  | **Correct poses (%)** | **Experimental ratio** |  | **Correct poses (%)** | | **Experimental ratio** | |  |  |
| TITGQKR | 15.84 | 0.10 * |  | 11.88 | | 0.16 | |  |  |
| LVTDLTK | 5.94 | 0.83 |  | 7.92 | | 0.29 | |  |  |
| GAAAGQR | 7.00 | 0.56 |  | 5.94 | | 0.83 | |  |  |
| FAATSFR | 12.87 | 0.10 * |  | 12.87 | | 0.24 | |  |  |
| VDPVNFK | 1.98 | 1.05 |  | 12.87 | | 0.10 * | |  |  |
| SALGEPKK | 6.93 | 1.04 |  | 1.98 | 0.94 | | | |  |
| Peptide sequences and experimental ratios were selected from table 5 of good and weak substrates for CPD domains I and II. *For experimental values lower than 0.10 (< 0,1), we assigned an exact value of 0.1. | | | | | | |  |  |  |
